# Engineering Fe–Ni Dual-Atom Sites Via Ru Nanoclusters on 3D Carbon Aerogel for Enhanced Bifunctional Oxygen Electrocatalysis

**DOI:** 10.1007/s40820-026-02211-x

**Published:** 2026-06-18

**Authors:** Yifan Zhang, Kexin Kong, Hongyuan Jie, Xiaoyan Jin, Long Tian, Yipu Liu, Zhijuan Pan, Seong-Ju Hwang, Liang Li, Zhe Wang

**Affiliations:** 1 College of Textile and Clothing Engineering, National Engineering Laboratory for Modern Silk, Soochow University, Suzhou, 215123 People’s Republic of China; 2 State Key Laboratory of Marine Resource Utilization in South China Sea, School of Materials Science and Engineering, Hainan University, Haikou, 570228 People’s Republic of China; 3 Department of Applied Chemistry, University of Seoul, Seoul, 02504 Republic of Korea; 4 Newtech Textile Technology Development (Shanghai) Co., Ltd., Shanghai, 201506 People’s Republic of China; 5 Department of Materials Science and Engineering, Yonsei University, Seoul, 03722 Republic of Korea; 6 School of Physical Science and Technology, Jiangsu Key Laboratory of Frontier Material Physics and Devices, Suzhou Key Laboratory of Intelligent Photoelectric Perception, Jiangsu Key Laboratory of Advanced Negative Carbon Technologies, Center for Energy Conversion Materials & Physics (CECMP), Soochow University, Suzhou, 215006 People’s Republic of China

**Keywords:** Dual-atom catalysts, Nanoclusters, Porous carbon aerogels, ORR/OER, Zinc–air batteries

## Abstract

**Highlights:**

- A heterogeneous catalyst consisting of atomically dispersed FeN_4_ and NiN_4_ sites along with Ru_6_ nanoclusters anchored on a 3D carbon aerogel was synthesized.
- The Ru_6_ nanoclusters optimize the electron transfer from the Fe/Ni centers to the key intermediate (*OH) at the rate-determining steps.
- Zin–air battery employed FeN_4_–Ru_6_–NiN_4_@PCA as the air cathode catalysts achieves an exceptional long-term cycling stability for over 2000 h.

**Abstract:**

Dual-atom catalysts (DACs) show great promise in catalyzing oxygen reduction/evolution reactions (ORR/OER), yet facing significant challenges in achieving simultaneous high catalytic activity and stability in zinc–air batteries (ZABs). In this study, we synthesized a porous three-dimensional carbon aerogel anchored with atomically isolated FeN_4_/NiN_4_ dual sites and Ru_6_ nanoclusters (FeN_4_–Ru_6_–NiN_4_@PCA) to address these challenges. The adjacent Ru_6_ nanoclusters effectively regulate the geometric structures of FeN_4_ and NiN_4_ sites and catalyze the formation of a highly graphitic carbon matrix. These structural features endow FeN_4_–Ru_6_–NiN_4_@PCA with remarkable ORR/OER activity and stability, outperforming counterparts with only FeN_4_/NiN_4_ dual species and benchmark Pt/C and RuO_2_ catalysts. Density functional theory calculations reveal that Ru6 clusters induce obvious electron redistribution of FeN_4_/NiN_4_ sites and optimize their electron transfer to the key oxygen intermediates (OH*) at the rate-determining steps, thereby accelerating the ORR and OER kinetics. When employed FeN_4_–Ru_6_–NiN_4_@PCA as the cathode catalyst in ZABs, the resulting ZAB delivers a peak power density of 197.76 mW cm^–2^ and demonstrates outstanding cycling stability over 2000 h, highlighting its great potential for use in applications of energy storage device.

**Graphical Abstract:**

**Figure:**
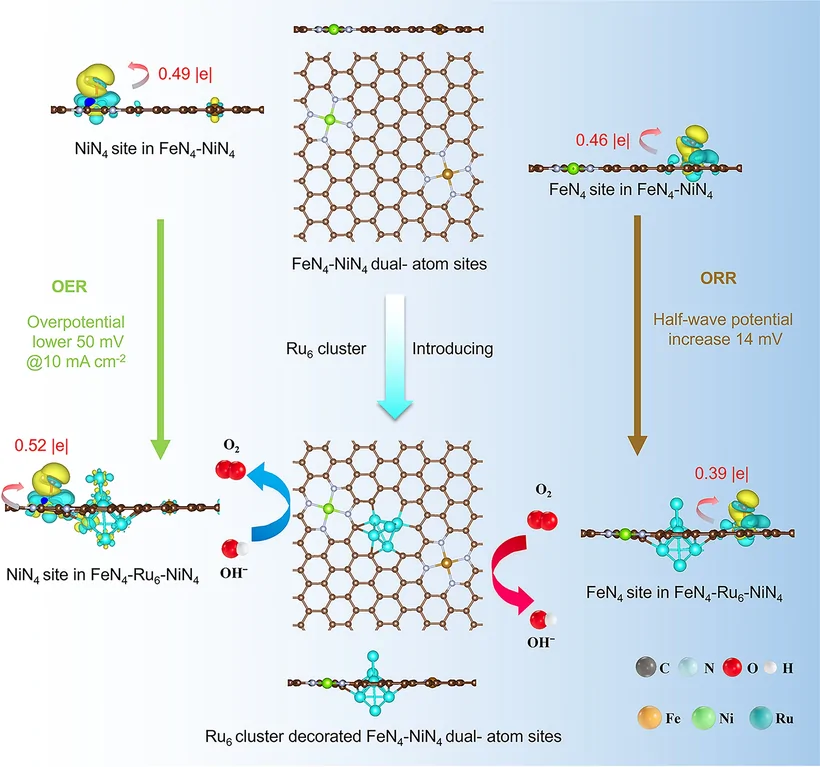

**Supplementary Information:**

The online version contains supplementary material available at 10.1007/s40820-026-02211-x.

## Introduction

The development of efficient renewable energy storage and conversion technologies is essential for addressing the global energy crisis and climate change. Zinc–air batteries (ZABs) have received significant attention owing to their high theoretical energy density, safety features, and economic advantage[1–4]. However, practical implementation faces challenges from the slow kinetics of the oxygen reduction reaction/oxygen evolution reaction (ORR/OER), along with poor reversible stability at air electrodes [5–7]. These issues collectively result in low energy conversion efficiency and limited cycling life. Recently, single-atom catalysts (SACs) have sparked tremendous research activity as valuable alternatives to commercial Pt/C and RuO_2_ catalysts for ORR/OER due to their adjustable electronic structures and high atomic utilization efficiency [3, 8–10]. However, SACs typically consist of only one type of metal active site, posing a significant challenge in achieving excellent catalyst functionality and durability for both the ORR and OER [11, 12].

Dual-atom catalysts (DACs) having two distinct metal active centers and various local configurations present a promising approach to address the limitations of SACs [9, 13–15]. DACs offer diverse active sites, adjustable local geometric structures (e.g., metal species, coordination environments, and spatial architectures), and potential synergistic effects between the metal sites [16, 17]. The structural flexibility of DACs enables precise optimization of the adsorption/desorption behavior of oxygen-containing intermediate species, thereby enhancing ORR and OER kinetics [18, 19]. Recent efforts have focused on developing carbon-supported DACs for bifunctional oxygen catalysis [20]. For instance, Tang et al. reported a Janus dual-atom catalyst on carbon nanosheets where Fe and Co sites were coordinated with N and O atoms, respectively. The strong interaction between the Fe–N_3_ and Co–O_3_ moieties can modulate the d-orbital energy level of the active metal atoms to optimize the binding energy of oxygenated intermediates and improving both ORR and OER performance [13].

In ZABs, alongside activity, stability is crucial for air cathode catalysts. Carbon-supported DACs often face challenges like metal leaching and carbon corrosion under high-voltage oxidizing conditions, particularly during high-current density charging [21–23]. Recent studies have shown that incorporating nanoclusters (NCs) or nanoparticles (NPs) into SACs/DACs on carbon substrates can notably enhance their electrochemical stability and corrosion resistance. In a prior study, a carbon fiber-supported DAC was developed with CoN_4_–FeN_4_ dual sites and Co_2_Fe–Fe_5_ nanoclusters coexisting. The synergy between the dual-atom sites and nanoclusters optimized the electronic structure of the metal active sites, reinforcing their interaction with the carbon supports [24, 25]. This optimization led to improved bifunctional oxygen catalytic activity and stability. However, most reported DACs only involve two types of metal species (e.g., A–B dual-atom sites with AB, A, or B clusters) due to challenges in precisely constructing dual-atom sites and nanoclusters with different metal species. To date, DACs combining A–B dual-atom sites with C-type nanoclusters (where C is a different metal) have been rarely documented, and their catalytic mechanism remains unexplored [26–28].

In this study, we synthesized a heterogeneous catalyst consisting of spatially separated FeN_4_ and NiN_4_ sites along with Ru_6_ nanoclusters supported on a three-dimensional (3D) porous carbon aerogel (FeN_4_–Ru_6_–NiN_4_@PCA). This catalyst exhibits outstanding bifunctional oxygen catalytic performance, with a half-wave potential of 0.874 V for the ORR and a potential of 1.58 V to reach a current density of 10 mA cm^−2^ for the OER. It surpassed not only catalysts containing only FeN_4_/NiN_4_ dual sites but also the benchmark materials Pt/C and RuO_2_. Moreover, the heterogeneous catalyst exhibits superior stability in both ORR and OER. Density functional theory (DFT) calculations indicated that the Ru6 clusters induce obvious charge redistribution of FeN_4_/NiN_4_ sites and optimize the electron transfer from the Fe/Ni centers to the key intermediate (*OH) at the rate-determining steps, thereby improving the ORR and OER kinetics. Notably, ZABs utilizing FeN_4_–Ru_6_–NiN_4_@PCA as the air cathode catalysts show a maximum power density of 197.76 mW cm^−2^ and sustained long-term cycling stability exceeding 2000 h (2000 cycles) [29]. Moreover, a system of three interconnected solid-state flexible ZABs successfully charged mobile phones and powered LED lights, showcasing significant promise for practical energy storage technologies.

## Experimental Section

### Materials

Carboxylated cellulose nanofibers (6 wt%) and ruthenium chloride hydrate (RuCl_3_·H_2_O, 98 wt%) were purchased from Shanghai Macklin Biochemical Co., Ltd. 2-Methylimidazole (C_4_H_6_N_2_, 98 wt%), nickel nitrate hexahydrate (Ni(NO_3_)_2_·6H_2_O, 98 wt%) and zinc nitrate hexahydrate (Zn(NO_3_)_2_·6H_2_O, 99.99 wt%) were all purchased from Shanghai Aladdin Biochemical Technology Co., Ltd. Iron(III) nitrate nonahydrate (Fe(NO_3_)_3_·9H_2_O, 99.99 wt%) was purchased from ‌Shanghai Acmec Biochemical Technology Co., Ltd‌. Graphene oxide (GO, 2 wt%) was purchased from Newtech Textile Technology Development (Shanghai) Co., Ltd..

### Synthesis of FeN_4_-Ru_6_-NiN_4_@PCA

#### Synthesis of Fe-ZIF@CNF

Five grams of carboxylated cellulose solution was dispersed in 10 g of deionized (DI) water and stirred with a magnetic stirrer to obtain a homogeneous dispersion. Subsequently, 0.1 g of zinc nitrate hexahydrate (Zn(NO_3_)_2_·6H_2_O) and 6.7 mg of iron nitrate hexahydrate (Fe(NO_3_)_3_·6H_2_O) (with a mass ratio of) were added into above dispersion, followed by stirring for 30 min, to form solution A. Separately, 1.6 g of 2-methylimidazole was dissolved in 15 g of DI water under magnetic stirring for 30 min, denoted as Solution B. Solution B was then poured into Solution A and stirred for 4 h. The mixture was centrifuge and washed with DI water to collect a pale-yellow product, denoted as Fe-ZIF@CNF.

#### Synthesis of NiRu@GO

0.3125 g of 2 wt% aqueous dispersion of graphene oxide (GO) was added in 15 g of DI water and subjected to low-temperature sonication with stirring. 4.2 mg of Ni(NO_3_)_2_·6H_2_O and 1.7 mg of RuCl_3_·H_2_O was then dissolved in above solution, followed by low-temperature stirring and sonication for 1 h, yielding NiRu@GO.

#### ***Synthesis of FeN***_***4***_***-Ru***_***6***_***-NiN***_***4***_***@PCA ***

The as-prepared Fe-ZIF@CNF was added into the NiRu@GO solution and stirred for 30 min to achieve a uniform mixture. The composite was then subjected to directional freeze-drying, resulting in the Fe-ZIF@CNF/NiRu@GO aerogel. The aerogel was transferred to a tube furnace for a two-step heat treatment under a flowing argon (Ar) atmosphere. The pyrolysis procedure was as following: The aerogel was firstly stabilized by heating from room temperature to 550 °C at 2 °C min^−1^, followed by a 2—h hold and then suffered from the carbonization treatment by heating from 550 to 920 °C at 5 °C min^−1^, followed by another 2 h hold. The final product was denoted as FeN_4_-Ru_6_-NiN_4_@PCA*.*

For comparison, reference samples including PCA, FeN_4_@PCA, and FeN_4_-NiN_4_@PCA were prepared using identical procedures. PCA was synthesized without adding any metal species, FeN_4_@PCA was prepared with only iron addition, and FeN_4_-NiN_4_@PCA was obtained with only iron and nickel species additions.

## Results and Discussion

### Morphological, Structural, and Electronic Features of FeN_4_–Ru_6_–NiN_4_@PCA

**Fig. 1:**
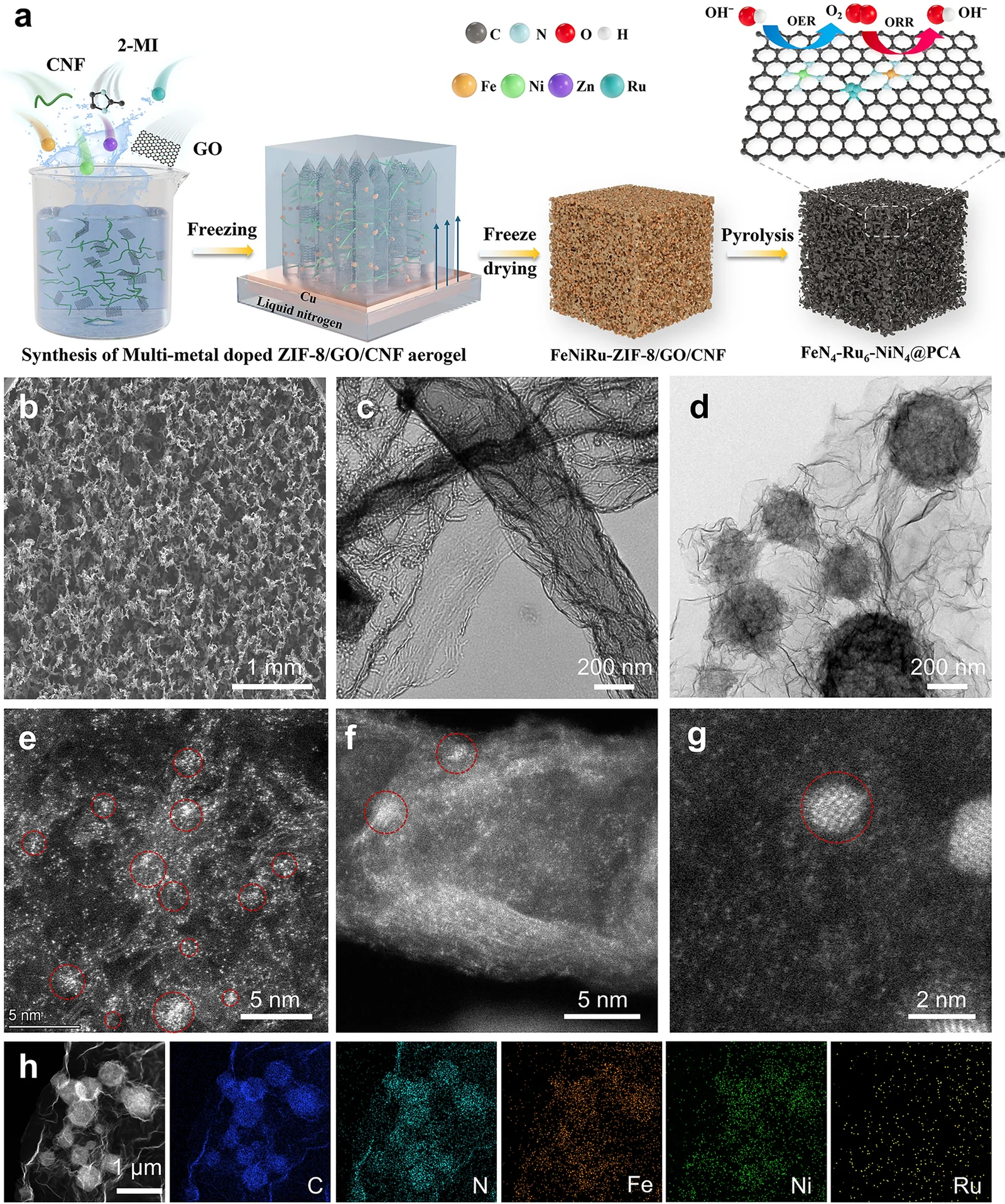
Morphology and structure of FeN_4_–Ru_6_–NiN_4_@PCA. **a** Synthetic procedure of the porous carbon aerogel with dual-atom NiN_4_/FeN_4_ sites and Ru_6_ NCs. **b** SEM, **c, d** TEM, and **e–g** AC-STEM images of FeN_4_–Ru_6_–NiN_4_@PCA. **h** HAADF-TEM image and EDS-elemental mapping data of FeN_4_–Ru_6_–NiN_4_@PCA

Figure 1a shows the synthesis process of FeN_4_–Ru_6_–NiN_4_@PCA. Atomically isolated dual NiN_4_/FeN_4_ sites accompanied with adjacent Ru_6_ NCs were stabilized on a porous 3D carbon aerogel through freeze-drying and subsequent pyrolysis. Initially, hydroxy cellulose fibers are dispersed in deionized water, followed by the addition of zinc nitrate and ferric nitrate to create a uniform solution. A 2-methylimidazole solution was then introduced, and the mixture was stirred for 4 h [30]. Subsequently, aqueous solutions containing Ni, Ru, and graphene oxide (GO) were added with continuous stirring. The resulting mixture underwent directional freeze-drying for 48 h to yield the FeNiRu-ZIF-8/GO/CNF aerogel. Finally, the aerogel was pyrolyzed under flowing Ar to form a 3D porous honeycomb structure (Fig. S1). During pyrolysis, cellulose fibers acted as the “skeleton,” and GO served as “cement,” transforming into carbon fibers and graphene, respectively. ZIFs converted into porous carbon nanocages entwined with carbon nanotubes (CNTs), collectively forming the porous carbon matrix [31]. Meanwhile, Fe and Ni species transitioned into well-dispersed NiN_4_ and FeN_4_ sites, respectively, and Ru species were reduced to form Ru_6_ NCs [32].

Scanning electron microscopy (SEM) images of FeN_4_–Ru_6_–NiN_4_@PCA (Fig. 1b) reveal a 3D honeycomb network structure with interconnected directional walls forming a highly porous matrix. Transmission electron microscopy (TEM) analysis was also conducted, as shown in Figs. 1c, d, and S2; FeN_4_–Ru_6_–NiN_4_@PCA exhibited a hierarchical structure, where MOF-derived porous carbon spheres, cellulose-derived carbon fibers, and several CNTs were loaded onto the reduced graphene oxide (rGO) sheet [33, 34]. Additionally, small nanoparticles are visible on the rGO sheets. N_2_ adsorption–desorption isotherm measurements were performed for FeN_4_–Ru_6_–NiN_4_@PCA to evaluate their Brunauer–Emmett–Teller (BET) surface area as well as pore size distribution. As plotted in Fig. S3, FeN_4_–Ru_6_–NiN_4_@PCA exhibited a huge specific surface area of 472.32 m^2^ g^–1^ and a type-IV isotherm, indicating the coexistence of micropores and mesopores. The pore size distribution curve confirms the micro–mesopore domain structure of FeN_4_–Ru_6_–NiN_4_@PCA, enhancing active site exposure and mass transfer during the ORR/OER process [24, 35]. The high-resolution transmission electron microscopy (HR-TEM) characterization data of the FeN₄–Ru₆–NiN₄@PCA catalyst (Fig. S4) indicate that there are no significantly disordered graphite layers in the porous carbon matrix. X-ray diffraction (XRD) patterns of PCA, FeN_4_@PCA, FeN_4_–NiN_4_@PCA, and FeN_4_–Ru_6_–NiN_4_@PCA (Fig. S5) display two diffusive reflections at ~ 26.2° and ~ 43.3°, corresponding to the (002) and (101) facets of the graphite lattice, respectively, without crystalline-phase peaks of Fe and Ni [36]. Furthermore, no crystalline-phase peak of Ru is detected, likely due to the small size (~ 2.5 nm) of the Ru nanoclusters (Fig. S6).

Raman spectroscopy (Fig. S7) reveals that FeN_4_–Ru_6_–NiN_4_@PCA has an I_D_/I_G_ ratio of 0.885, significantly lower than those of PCA (0.928), FeN_4_@PCA (0.923), and FeN_4_–NiN_4_@PCA (0.895), indicating that the incorporation of FeN_4_–NiN_4_ dual sites and Ru clusters substantially enhances the graphitization of the carbon matrix. The highly graphitized structure of FeN_4_–Ru_6_–NiN_4_@PCA promotes charge transfer and improves the durability of the carbon substrate against corrosion[16]. Figure 1e–g**,** S8 and S9 present the aberration corrected-high angle annular dark-field scanning TEM (AC HAADF-STEM) data of FeN_4_–Ru_6_–NiN_4_@PCA in various selection fields, including ZIFs-derived carbon nanocage and CNTs, as well as the rGO. These images revealed that abundant isolated bright spots, corresponding to Fe/Ni SACs and several Ru NCs, are uniformly distributed on the carbon matrix. The HAADF-STEM image (Fig. 1h) and the energy-dispersive spectroscopy (EDS)-mapping data indicated a uniform distribution of C, N, Fe, Ni, and Ru species in the entire region of FeN_4_–Ru_6_–NiN_4_@PCA, confirming the coexistence of isolated Ni and Fe atoms along with Ru NCs on the porous nitrogen-doped porous carbon aerogel [37].

**Fig. 2:**
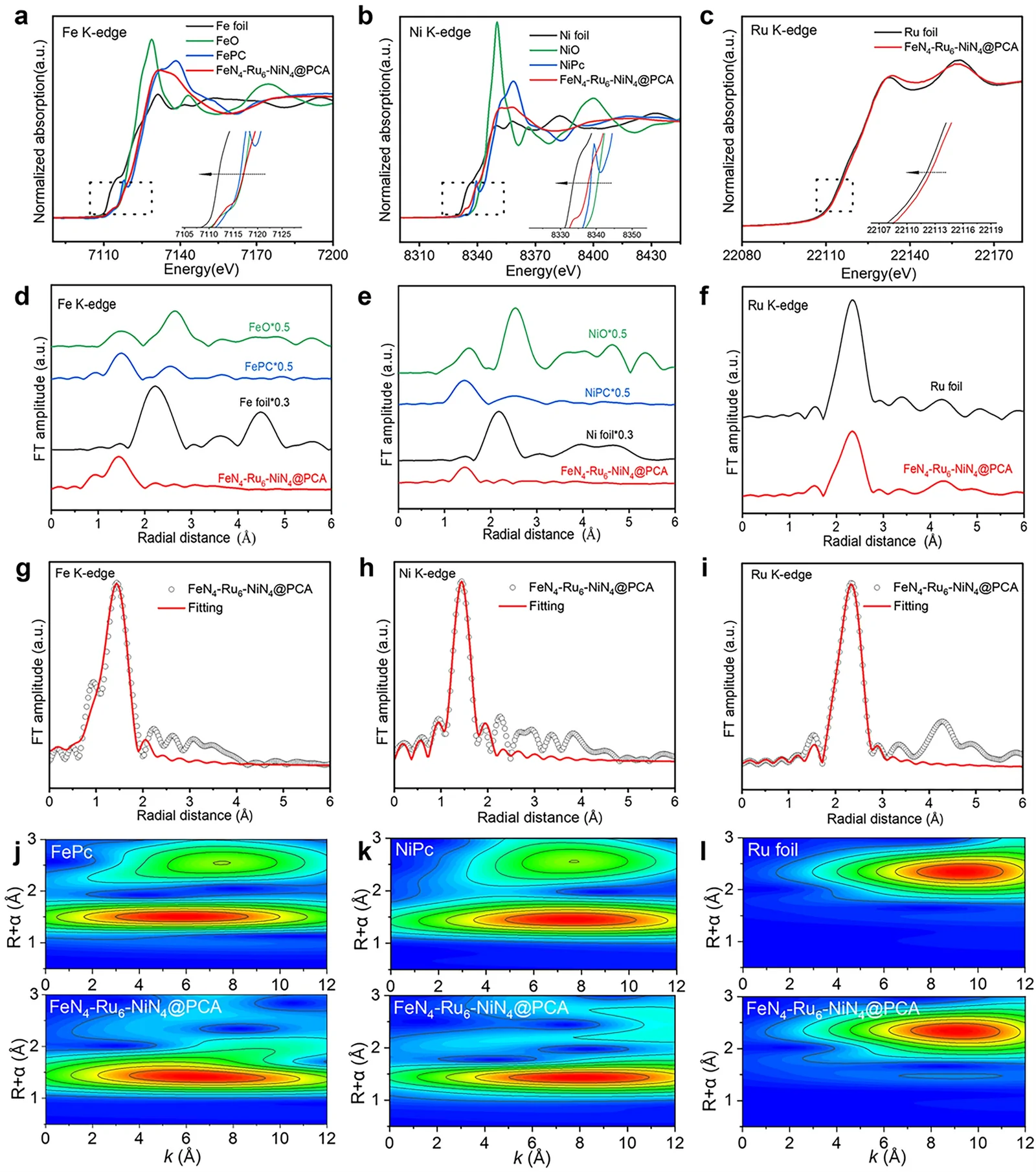
Valence state and atomic local structure of FeN_4_–Ru_6_–NiN_4_@PCA. **a–c** XANES, **d–f** FT-EXAFS, **g–i** Measured and fitted FT-EXAFS spectra, and **j–l** WT data of the *k*^3^-weighted EXAFS spectra of FeN_4_–Ru_6_–NiN_4_@PCA and the references

X-ray absorption spectroscopic analyses were performed to determine the valence states and local atomic structures of the Fe, Ni, and Ru species in FeN_4_–Ru_6_–NiN_4_@PCA. Reference spectra for Ni, Fe, and Ru foils, NiO, FeO, RuO_2_, and nickel phthalocyanine (NiPc)/iron phthalocyanine (FePc) were also recorded for comparison. As depicted in Fig. 2a, the X-ray absorption near-edge structure (XANES) spectrum of FeN_4_–Ru_6_–NiN_4_@PCA at Fe K-edge resembles those of FePc and FeO, indicating a cationic valence state for the Fe species in FeN_4_–Ru_6_–NiN_4_@PCA. The Fe K-edge Fourier transformed-extended X-ray absorption fine structure (FT-EXAFS) spectrum of FeN_4_–Ru_6_–NiN_4_@PCA shows a prominent peak at R =  ~ 1.47 Å (Fig. 2d), similar to the Fe–N-related FT feature of FePc, suggesting the presence of an Fe–N coordination shell. Wavelet transform (WT) analysis was conducted on the EXAFS data to identify atomic arrangements in R- and k-spaces. In the Fe K-edge WT data for FeN_4_–Ru_6_–NiN_4_@PCA (Figs. 2j and S10), a clear peak from the Fe–N bond is visible at ~ 6.5 Å^−1^ in k-space, similar to that of FePc. The EXAFS fitting analysis (Fig. 2g and Table S1) demonstrated that the Fe–N coordination shell in FeN_4_–Ru_6_–NiN_4_@PCA shows the coordination number of 3.94, highlighting that isolated Fe atom coordinates with four N ligands to form FeN_4_ units.

As presented in Fig. 2b, the Ni K-edge XANES analysis indicated that the edge jump of Ni in FeN_4_–Ru_6_–NiN_4_@PCA falls between that of Ni metal foil and Ni^2+^O reference, suggesting a positive valence state of the Ni species. The Ni K-edge energy in FeN_4_–Ru_6_–NiN_4_@PCA closely matched that of the reference NiPc, implying similar oxidation states for the Ni cations in both materials. The EXAFS spectrum of FeN_4_–Ru_6_–NiN_4_@PCA at Ni K-edge exhibited a prominent FT peak at R =  ~ 1.44 Å (Fig. 2e), corresponding to the Ni–N bonding pair, similar to the Ni–N-related peak in NiPc, indicating exclusive coordination of Ni atoms with N atoms. Additionally, the WT contour data in Figs. 2k and S11 revealed a distinct peak related to the Ni–N coordination pair at ~ 7.5 Å^−1^ for FeN_4_–Ru_6_–NiN_4_@PCA, closely resembling NiPc, thus confirming Ni–N coordination. Curve-fitting analysis of the EXAFS data yielded the quantitative local structural parameters of FeN_4_–Ru_6_–NiN_4_@PCA. As shown in Fig. 2h and Table S1, the Ni–N bonding pair in FeN_4_–Ru_6_–NiN_4_@PCA has the coordination number of 3.81, indicating coordination of each isolated Ni atom with four N atoms, confirming the presence of Ni–N_4_ moieties.

XANES analysis of the Ru K-edge showed a positive shift compared to that of the Ru foil (Fig. 2c). The FT-EXAFS spectrum of the Ru K-edge for FeN_4_–Ru_6_–NiN_4_@PCA displayed a prominent peak at R =  ~ 2.34 Å (Fig. 2f), nearly identical to that of the Ru foil. The WT analysis of the EXAFS data for FeN_4_–Ru_6_–NiN_4_@PCA resembled that of the Ru foil (Fig. 2l). The EXAFS spectrum fitting analysis (Fig. 2i and Table S1) revealed that the coordination number of Ru–Ru in FeN_4_–Ru_6_–NiN_4_@PCA is 5.34, significantly lower than that of the Ru foil, indicating the presence of Ru_6_ NCs. In summary, FeN_4_–Ru_6_–NiN_4_@PCA was identified as a heterostructured catalyst with dual FeN_4_/NiN_4_ sites, in addition to Ru_6_ NCs.

X-ray photoelectron spectroscopy (XPS) was utilized to characterize the surface elemental compositions and valence states of the synthesized materials. Figure S12 shows the full XPS survey spectra of FeN_4_-Ru_6_-NiN_4_@PCA and FeN_4_-NiN_4_@PCA.As presented in Fig. S13, the Fe 2*p* XPS datum of FeN_4_–Ru_6_–NiN_4_@PCA exhibits distinct peaks at 711.2 eV (Fe^2+^),723.7 eV (Fe^2+^), 713.6 eV (Fe^3+^), and 726.9 eV (Fe^3+^), confirming the absence of zero-valent Fe species [38]. Ni 2*p* XPS measurements on FeN_4_–Ru_6_–NiN_4_@PCA reveal peaks at 853.2 eV (Ni^2+^) and 870.6 eV (Ni^2+^), assigned as the Ni 2*p*_3/2_ and Ni 2*p*_1/2_ states of Ni cations, respectively (Fig.s S14) [39]. The N 1* s* XPS data of FeN_4_–Ru_6_–NiN_4_@PCA in Fig. S15 were deconvoluted into five components: pyridinic N (398.4 eV), metal–nitrogen (399.3 eV), pyrrolic N (400.3 eV), graphitic N (401.2 eV), and oxidized N (402.9 eV). The observation of a peak at 399.3 eV confirms the presence of Fe/Ni–N bonds in FeN_4_–Ru_6_–NiN_4_@PCA [40]. Additionally, due to its low Ru content, no significant XPS signal of Ru was detected in FeN_4_–Ru_6_–NiN_4_@PCA (Fig. S16). The inductively coupled plasma mass spectroscopy (ICP-MS) measurement was conducted to quantify the metal content in FeN_4_–Ru_6_–NiN_4_@PCA. InTable S2, the contents of Fe, Ni, and Ru were 0.82, 0.74, and 0.39 wt%, respectively.

### Oxygen Electrocatalyst Performance of FeN_4_–Ru_6_–NiN_4_@PCA

**Fig. 3:**
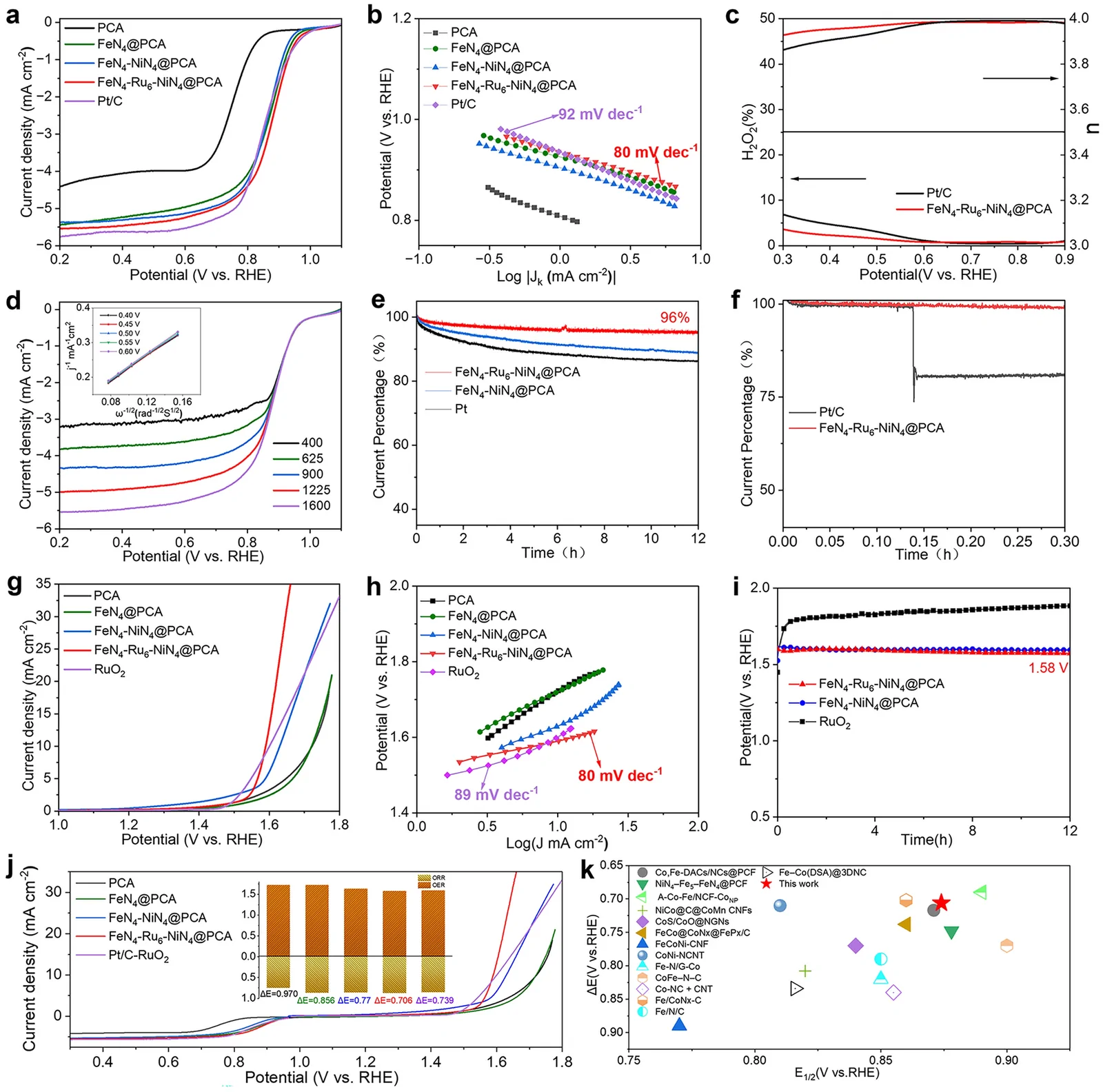
Electrocatalytic ORR/OER performance and stability of FeN_4_–Ru_6_–NiN_4_@PCA.** a** ORR-LSV curves at 1600 rpm for PCA, FeN_4_@PCA, FeN_4_-NiN_4_@PCA, FeN_4_–Ru_6_–NiN_4_@PCA, and Pt/C catalysts. **b** Tafel plots for FeN_4_–Ru_6_–NiN_4_@PCA and the references. **c** H_2_O_2_ yield and electron transfer number (n) for FeN_4_–Ru_6_–NiN_4_@PCA and Pt/C catalysts. **d** LSV data of FeN_4_–Ru_6_–NiN_4_@PCA at various rotation speeds; inset presents the corresponding K–L plot. **e** Normalized chronoamperometry data of FeN_4_–Ru_6_–NiN_4_@PCA, and Pt/C catalysts at 0.6 V vs. RHE. **f** Methanol tolerance curves of FeN_4_–Ru_6_–NiN_4_@PCA and Pt/C catalysts during the chronoamperometric test. **g** OER-LSV curves at 1600 rpm for PCA, FeN_4_@PCA, FeN_4_–NiN_4_@PCA, FeN_4_–Ru_6_–NiN_4_@PCA, and Pt/C catalysts. **h** Tafel slopes for FeN_4_–Ru_6_–NiN_4_@PCA and the references. **i** Chronopotentiometric curve of OER functionality of FeN_4_–Ru_6_–NiN_4_@PCA, FeN_4_–NiN_4_@PCA, and RuO_2_ at 10 mA cm^–2^. **j** ORR-OER-LSV curves of FeN_4_–Ru_6_–NiN_4_@PCA, and the references. **k** Comparison data of the *E*_1/2_ and Δ*E* for FeN_4_–Ru_6_–NiN_4_@PCA and other recently reported catalysts

The electrocatalytic ORR/OER activities of FeN_4_–Ru_6_–NiN_4_@PCA were assessed using a standard three-electrode cell with a 0.1 M KOH electrolyte. Reference samples included PCA, FeN_4_@PCA, FeN_4_–NiN_4_@PCA, as well as benchmark Pt/C and RuO_2_ catalysts. All collected voltages were compared to that of reversible hydrogen electrode. In Fig. 3a, the linear sweep voltammetry (LSV) datum of FeN_4_–Ru_6_–NiN_4_@PCA displays a high half-wave potential (E_1/2_) of 0.874 V, outperforming Pt/C (E_1/2_, 0.850 V), PCA (0.75 V), FeN_4_@PCA (0.864 V), and FeN_4_–NiN_4_@PCA (0.860 V) [41]. As illustrated in Fig. 3b, the Tafel slope of FeN_4_–Ru_6_–NiN_4_@PCA is 80 mV dec^−1^, which is lower than those of Pt/C (92 mV dec^−1^), PCA (104 mV dec^−1^), and FeN_4_–NiN_4_@PCA (85 mV dec^−1^), indicating faster ORR kinetics. Rotating ring-disk electrode measurements were conducted. In Fig. 3c, the H_2_O_2_ yield of FeN_4_–Ru_6_–NiN_4_@PCA remains below 6.9% in the potential range of 0.3–0.9 V, with electron transfer numbers in the range of 3.86–3.99, suggesting that FeN_4_–Ru_6_–NiN_4_@PCA follows a four-electron ORR reaction pathway. This finding aligns with the Koutecky–Levich (K–L) plots from LSV data at different rotation speeds (Fig. 3d) [42].

The ORR stability of FeN_4_–Ru_6_–NiN_4_@PCA was assessed through a chronoamperometric (i–t) examination at a potential of 0.60 V. As displayed in Fig. 3e, FeN_4_–Ru_6_–NiN_4_@PCA retained 96% of its initial current density after 12 h of ORR process. In contrast, FeN_4_–NiN_4_@PCA preserved 89% of its initial current, whereas Pt/C retained 86% under the same conditions. Morover, a slight negative shift in the polarization curves of FeN_4_–Ru_6_–NiN_4_@PCA, corresponding to a 9 mV loss of E_1/2_, is observed after 20,000 CV cycles (Fig. S17). In contrast, the E_1/2_ degradation for Pt/C is 21 mV under the same conditons. These findings emphasize the outstanding stability of FeN_4_–Ru_6_–NiN_4_@PCA during the ORR. This enhanced stability can be attributed to the significant coupling effect between the dual-atom sites and the NCs, which reduces demetallation of the metal sites and enhances the corrosion resistance of the carbon support. Furthermore, as depicted in Fig. 3f, FeN_4_–Ru_6_–NiN_4_@PCA displayed remarkable methanol tolerance, sustaining a consistent current after methanol introduction in chronoamperometric i–t tests. In contrast, benchmark Pt/C exhibited a significant decline in current density after the methanol injection [43].

For OER, as depicted in Fig. 3g, FeN_4_–Ru_6_–NiN_4_@PCA demonstrates a reduced potential of 1.58 V to reach a current density of 10 mA cm^–2^ (E_j=10_), surpassing FeN_4_–NiN_4_@PCA (E_j=10_, 1.63 V), FeN_4_@PCA (E_j=10_, 1.72 V), and PCA (E_j=10_, 1.72 V). This highlights the significant role of Ru NCs in fostering a synergistic effect between FeN_4_–NiN_4_ dual sites and Ru clusters, thereby enhancing their OER performance. In addition, FeN_4_–Ru_6_–NiN_4_@PCA exhibits a lower E_j=10_ compared to commercial RuO_2_ (E_j=10_, 1.59 V), affirming its superior OER activity. The Tafel slope of FeN_4_–Ru_6_–NiN_4_@PCA is 80 mV dec^−1^ (Fig. 3h), notably lower than PCA (145 mV dec^–1^), FeN_4_@PCA (122 mV dec^–1^), FeN_4_–NiN_4_@PCA (94 mV dec^–1^), and RuO_2_ (89 mV dec^–1^), demonstrating its enhanced OER kinetics. Additionally, as shown in Fig. 3i, the OER catalytic stability of FeN_4_–Ru_6_–NiN_4_@PCA surpasses that of RuO_2_. The FeN_4_–Ru_6_–NiN_4_@PCA catalyst exhibits a minimal negative shift (11 mV) at E_j=10_ after 3000 potential cycles (Fig. S18), further demonstrating its superior stability compared to RuO_2_ under the same conditions. The overall bifunctional electrocatalyst functionality of FeN_4_–Ru_6_–NiN_4_@PCA was evaluated by determining the difference between the E_j=10_ for the OER and E_1/2_ for the ORR, i.e., ΔE = E_j=10_ − E_1/2_. As plotted in Fig. 3j and Table S3, the ΔE value of FeN_4_–Ru_6_–NiN_4_@PCA was 0.706 V, significantly lower than PCA (ΔE = 0.970 V), FeN_4_@PCA (ΔE = 0.856 V), and FeN_4_–NiN_4_@PCA (ΔE = 0.770 V). The bifunctional oxygen catalytic performance of FeN_4_–Ru_6_–NiN_4_@PCA is comparable to, or even superior to, that of the most recently reported SAC/DACs (Fig. 3k). Hence, the exceptional bifunctional electrocatalyst performance and durability of FeN_4_–Ru_6_–NiN_4_@PCA position it as a promising candidate for renewable energy storage device applications [11, 44–46].

### Rechargeable ZAB Application of FeN_4_–Ru_6_–NiN_4_@PCA

**Fig. 4:**
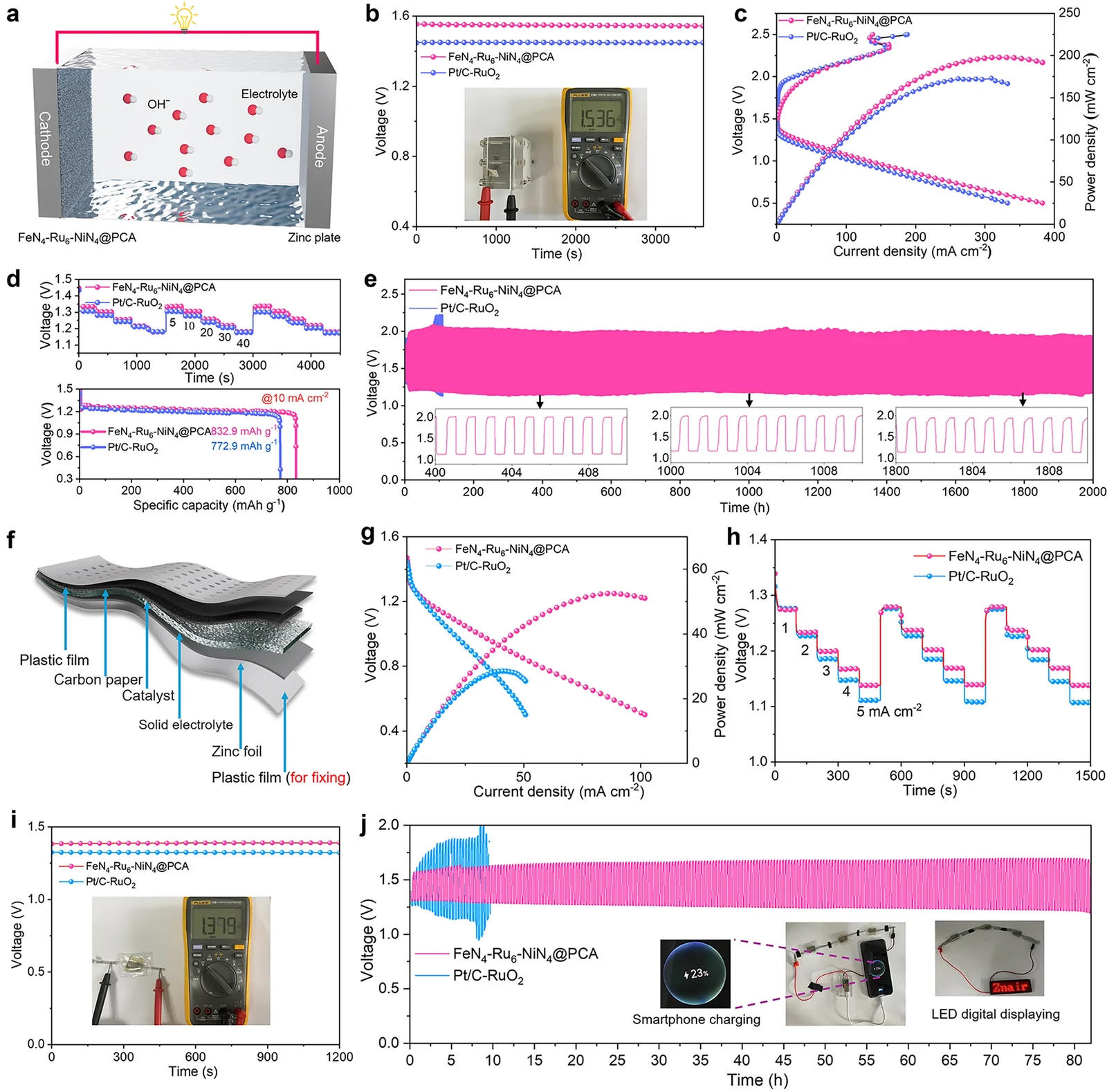
Performance of ZABs with FeN_4_–Ru_6_–NiN_4_@PCA as air cathode. **a** Scheme of liquid ZABs. **b** OCV (inset: OCV recorded by a multimeter), **c** polarization curves for charge–discharge and discharge power density, and **d** galvanostatic discharge curves of the ZABs at varying current densities (5–40 mA cm^−2^) (top), specific capacity curves at 10 mA cm^−2^ (bottom). **e** Galvanostatic long-term cycling durability at 2 mA cm^−2^ for the assembled liquid ZABs. **f** Schematic diagram of the flexible solid-state ZABs. **g** OCV, **h** discharge polarization and power density curves, **i** current density-dependent galvanostatic discharge curves, and **j** constant current charge–discharge cycle performance of the flexible solid-state ZABs at 1 mA cm^−2^

Given the outstanding ORR/OER functionalities of FeN_4_–Ru_6_–NiN_4_@PCA, we evaluated its practical applicability in ZABs. An in-house aqueous ZAB was constructed using FeN_4_–Ru_6_–NiN_4_@PCA deposited on carbon paper as the air cathode (see Figs. 4a and S19). For comparison, another ZAB was prepared by employing Pt/C + RuO_2_ as a cathode catalyst. As depicted in Fig. 4b, the open-circuit voltage (OCV) of the FeN_4_–Ru_6_–NiN_4_@PCA-based ZAB reaches 1.536 V, significantly higher than that of the Pt/C + RuO_2_-based homolog (1.450 V). As presented in Fig. 4c, the FeN_4_–Ru_6_–NiN_4_@PCA-based ZAB shows a reduced charge–discharge voltage gap compared with that of the Pt/C + RuO_2_-based homolog, indicating its outstanding bifunctional ORR and OER performance in practical ZABs. The FeN_4_–Ru_6_–NiN_4_@PCA-based ZAB achieved a peak power density of 197.76 mW cm^–2^, which surpassed that of the Pt/C + RuO_2_-based ZAB (173.2 mW cm^–2^). Additionally, as illustrated in Fig. 4d (top), the FeN_4_–Ru_6_–NiN_4_@PCA-based ZAB demonstrated improved discharge rate capability and stability at current densities ranging from 5 to 40 mA cm^–2^. Moreover, the galvanostatic discharge specific capacity of the FeN_4_–Ru_6_–NiN_4_@PCA-based ZAB was 832.9 mA h g^–1^ (Fig. 4d (bottom)), outperforming that of the Pt/C + RuO_2_-based ZAB (772.9 mA h g^−1^). To evaluate the cycling stability of FeN_4_–Ru_6_–NiN_4_@PCA in ZABs, galvanostatic cycling tests were carried out at a current density of 2 mA cm^−2^ with 30 min of discharge and 30 min of charge. Impressively, the FeN_4_–Ru_6_–NiN_4_@PCA-based ZAB demonstrated a minimal charge–discharge voltage gap and no noticeable voltage decay over 2000 h (Fig. 4e), indicating its exceptional long-term durability. In contrast, the charge–discharge potentials of the Pt/C + RuO_2_-based ZAB decreased significantly after 95 h of cycling. As shown in Fig. S20, the performance of FeN_4_–Ru_6_–NiN_4_@PCA-based ZAB competitively against those employing recently reported SACs/DACs [47–49]. These results emphasize the exceptional bifunctional oxygen electrocatalyst activity and stability of FeN_4_–Ru_6_–NiN_4_@PCA in liquid ZABs [50]. Moverover, FeN_4_–Ru_6_–NiN_4_@PCA also demonstrates outstanding structural robustness and stable chemical property after the cycling stability test, as evidenced by the TEM images in Fig. S21 and XRD pattern in Fig. S22.

Flexible solid-state ZABs were constructed by employing carbon paper loaded with FeN_4_–Ru_6_–NiN_4_@PCA powder as the flexible air cathode, the polished zinc foil as the anode, and a flexible polyacrylamide (PAM) organohydrogel ternary hydrogel as the solid-state electrolyte (Fig. 4f) [51–53]. In Fig. 4g, the FeN_4_–Ru_6_–NiN_4_@PCA-based flexible ZAB showed a higher OCV of 1.379 V than that of the Pt/C + RuO_2_-based flexible ZAB (1.32 V). Additionally, the FeN_4_–Ru_6_–NiN_4_@PCA-based flexible ZAB achieved a maximum power density of 53.4 mW cm^−2^, surpassing that of the Pt/C + RuO_2_-based flexible ZAB (Fig. 4h), and displayed excellent discharge rate performance at various current densities (1–5 mA cm^−2^) (Fig. 4i). Notably, the flexible ZAB also exhibited stable cycle performance up to 80 h at 1 mA cm^−2^ (Fig. 4j). Furthermore, three serially connected solid-state flexible ZABs could power a mobile phone and an LED light (Fig. 4j, inset), showcasing their wide potential for practical energy storage applications [54]. Furthermore, the flexible ZABs based on FeN_4_–Ru_6_–NiN_4_@PCA also exhibit good cycling stability at current densities of 2 and 5 mA cm^−2^ (Fig. S23).

### Theoretically Elucidated Mechanism for Catalytic Activity of FeN_4_-Ru_6_-NiN_4_@PCA

**Fig. 5:**
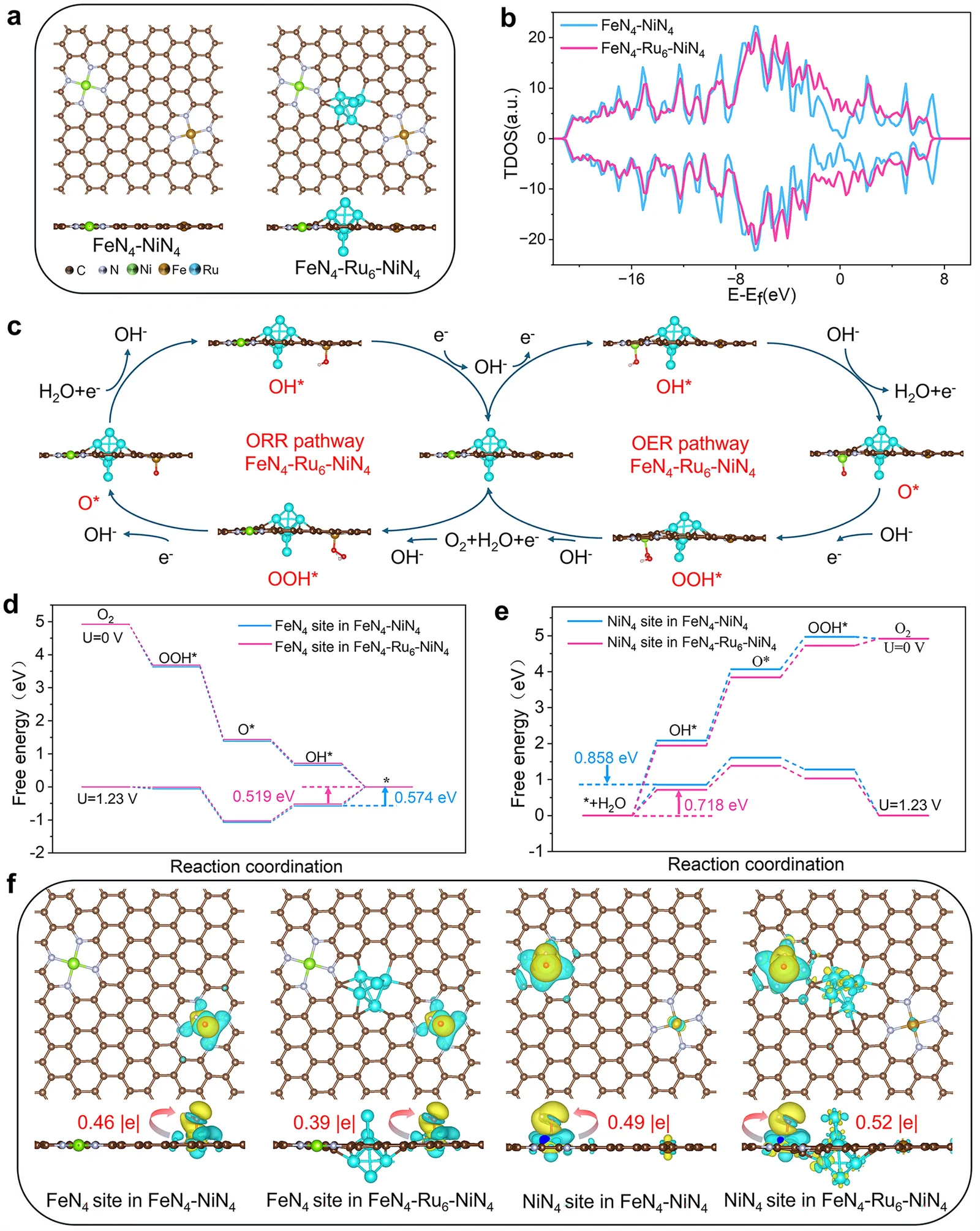
DFT calculation results of ORR and OER performance for the FeN_4_–Ru_6_–NiN_4_ and FeN_4_–NiN_4_. **a** Top and side views of three local atomic arrangements, **b** TDOS of FeN_4_–NiN_4_, and FeN_4_–Ru_6_–NiN_4_ models. **c** Adsorption geometries along the ORR/OER pathway on the FeN_4_ and NiN_4_ sites in the FeN_4_–Ru_6_–NiN_4_. **d** ORR free energy diagrams for FeN_4_ sites in FeN_4_–NiN_4_ and FeN_4_–Ru_6_–NiN_4_ at 0 and 1.23 V versus RHE. **e** OER free energy diagrams for NiN_4_ sites in FeN_4_–NiN_4_ and FeN_4_–Ru_6_–NiN_4_ at 0 and 1.23 V versus RHE. **e** Adsorption configurations at each key step on the FeN_4_ and Ru@FeN_4_ sites in the FeN_4_–Ru_6_–NiN_4_@PCA model. **f** Charge density difference of FeN_4_ and NiN_4_ sites in FeN_4_–Ru_6_–NiN_4_ and FeN_4_–NiN_4_ after OH adsorption (yellow contour: electron accumulation, blue contour: electron depletion)

To gain an in-depth understanding the enhancement mechanism of Ru nanoclusters on the ORR/OER performance of FeN_4_ and NiN_4_ sites, we performed density functional theory (DFT) calculations. Based on the experimental data, we constructed two atomic models: FeN_4_-Ru_6_-NiN_4_ and FeN_4_-NiN_4_ (Fig. 5a), in which FeN_4_-Ru_6_-NiN_4_ represents the active site in FeN_4_-Ru_6_-NiN_4_@PCA and FeN_4_-NiN_4_ acts as the active site in FeN_4_-NiN_4_@PCA. As shown in Fig. 5b, compared with FeN_4_-NiN_4_, FeN_4_-Ru_6_-NiN_4_ exhibits a higher total density of states (TDOS) near the Fermi level, indicating enhanced electronic conductivity, which facilitates charge transfer during the ORR/OER processes. Both the ORR and OER proceed via a four-electron pathway involving the oxygen-containing intermediates of *O₂, *OOH, *O, and *OH (Figs. 5c and S24), where Fe and Ni centers serve as the active sites for ORR and OER, respectively.

As shown in Fig. 5d, the Gibbs free energy diagrams of the ORR on FeN_4_ sites in both FeN_4_-Ru_6_-NiN_4_ and FeN_4_-NiN_4_ demonstrate that all electron transfer steps occur spontaneously at zero potential (U = 0 V). At U = 1.23 V, the FeN_4_ sites in FeN_4_-Ru_6_-NiN_4_ exhibits the largest energy difference at the fourth reaction step (*OH + e⁻ → * + OH⁻), indicating that the desorption of *OH from the Fe centers is the rate-determining step (RDS). The free energy barrier of RDS for FeN_4_-Ru_6_-NiN_4_ is calculated to be 0.519 eV, which is significantly lower than that for the FeN_4_ sites in FeN_4_-NiN_4_ (0.574 eV), suggesting that FeN_4_-Ru_6_-NiN_4_ species possesses thermodynamic advantages during the ORR. Furthermore, the charge density difference plots and Bader charge analysis of FeN_4_ sites in FeN_4_-Ru_6_-NiN_4_ and FeN_4_-NiN_4_ after O* absorption (Fig. 5f) demonstrates that the introduction of Ru_6_ clusters induce electron redistribution and fewer electron transfer from Fe to O for OH*, which facilitates the OH* desorption at RDS. These results indicate that introducing Ru_6_ nanoclusters in FeN_4_-Ru_6_-NiN_4_@PCA effectively weakens OH* adsorption on the FeN_4_ site, thus enhancing ORR kinetics.

We also conducted DFT calculations on NiN_4_ sites in both FeN_4_-Ru_6_-NiN_4_ and FeN_4_-NiN_4_ for the OER processes. As shown in Fig. 5e, at U = 0 V, the activation energy of the rate-determining step (RDS) of OER (OH^−^  + * → OH* + e^−^) at the FeN_4_-Ru_6_-NiN_4_ is 1.948 eV, which is significantly lower than the 2.088 eV at the FeN_4_-NiN_4_. At U = 1.23 V, the maximum energy consumption at both the FeN_4_-Ru_6_-NiN_4_ and FeN_4_-NiN_4_ sites also occurs at the first step. This indicates that the introduction of Ru_6_ clusters adjacent to the NiN_4_ sites can effectively reduce the reaction energy barrier and enhance the kinetics of the OER. Figure 5f shows the charge density difference distributions and Bader charge analysis results of NiN_4_ sites in FeN_4_-Ru_6_-NiN_4_ and FeN_4_-NiN_4_ after adsorbing the *OH intermediate. The results suggest that the presence of Ru_6_ clusters in FeN_4_-Ru_6_-NiN_4_ facilitate more electron (0.52 e) transferred from NiN_4_ site to OH compared to that of FeN_4_-NiN_4_ (0.49 e), which enhances the interactions between the NiN_4_ sites and OH and thus reduce the energy barrier of the RDS for OER processes. In summary, the Ru_6_ clusters adjacent NiN_4_ and FeN_4_ sites effectively modulate their geometric/electronic structures, which facilitates the charge transfer and optimizes the adsorption–desorption behaviors of the key intermediates at RDSs, and ultimately enhances the ORR and OER kinetics.

## Conclusions

In this study, we developed a porous carbon aerogel-supported heterogeneous catalyst containing atomically isolated dual NiN_4_/FeN_4_ sites associated with Ru_6_ nanoclusters (FeN_4_–Ru_6_–NiN_4_@PCA). Owing to the strong interactions between the Fe/Ni dual sites and Ru nanoclusters, FeN_4_–Ru_6_–NiN_4_@PCA demonstrates exceptional ORR/OER activity and stability, achieving a high half-wave potential of 0.874 V for the ORR and a low potential of 1.58 V at 10 mA cm^−2^ for the OER, surpassing benchmark Pt/C and RuO_2_ electrocatalysts. DFT calculations reveal that the Ru_6_ clusters induce obvious electron redistribution of FeN_4_/NiN_4_ active sites, which optimize the electron transfer from the Fe/Ni to the key intermediate (*OH) at the RDSs, thus promoting the ORR/OER kinetics. Notably, aqueous ZABs utilizing FeN_4_–Ru_6_–NiN_4_@PCA as the air cathode deliver a high peak power density of 197.76 mW cm^−2^ and stable cycling performance exceeding 2000 h (2000 cycles), significantly outperforming the ZAB with a Pt/C + RuO_2_ air cathode. Furthermore, serially assembled flexible solid-state ZABs can charge mobile phones and power LED lights. This work presents an effective approach for developing highly active and robust bifunctional electrocatalysts coupling with dual-atom sites and nanoclusters for practical energy storage technologies.

## Supplementary Information

Below is the link to the electronic supplementary material.

Supplementary file1 (DOCX 21700 KB)
