# Supplementary material for: Engineering Fe–Ni Dual-Atom Sites Via Ru Nanoclusters on 3D Carbon Aerogel for Enhanced Bifunctional Oxygen Electrocatalysis
Source: Nanomicro Lett. 2026 Jun 18;18:404. doi: 10.1007/s40820-026-02211-x (PMC13276091; doi:10.1007/s40820-026-02211-x)
Supplement: Supplementary file 1 — Supplementary file1 (DOCX 21700 KB) [file 40820_2026_2211_MOESM1_ESM.docx]

Supporting Information for

**Engineering Fe–Ni Dual-Atom Sites via Ru Nanoclusters on 3D Carbon Aerogel for Enhanced Bifunctional Oxygen Electrocatalysis**

Yifan Zhang^1^, Kexin Kong^1^, Hongyuan Jie^2^, Xiaoyan Jin^3^, Long Tian^4^, Yipu Liu^2^, Zhijuan Pan^1^, Seong-Ju Hwang^5,^ *, Liang Li^6,^ *, and Zhe Wang^1,^ *

^1^ College of Textile and Clothing Engineering, National Engineering Laboratory for Modern Silk, Soochow University, Suzhou 215123, P. R. China

^2^ State Key Laboratory of Marine Resource Utilization in South China Sea, School of Materials Science and Engineering, Hainan University, Haikou 570228 P. R. China

^3^ Department of Applied Chemistry, University of Seoul, Seoul 02504, Republic of Korea

^4^ Newtech Textile Technology Development (Shanghai) Co., Ltd., Shanghai 201506, P. R. China

^5^ Department of Materials Science and Engineering, Yonsei University, Seoul 03722, Republic of Korea

^6^ School of Physical Science and Technology, Jiangsu Key Laboratory of Frontier Material Physics and Devices, Suzhou Key Laboratory of Intelligent Photoelectric Perception, Jiangsu Key Laboratory of Advanced Negative Carbon Technologies, Center for Energy Conversion Materials & Physics (CECMP), Soochow University, Suzhou 215006, P. R. China

*Corresponding authors. E-mail: [zhewang1119@suda.edu.cn](mailto:zhewang1119@suda.edu.cn) (Zhe Wang); [hwangsju@yonsei.ac.kr](mailto:hwangsju@yonsei.ac.kr) (Seong-Ju Hwang); [lli@suda.edu.cn](mailto:lli@suda.edu.cn) (Liang Li)

S1 Experimental Section

**S1.1 Characterizations**

The obtained materials were subjected to X-ray powder diffraction (XRD) measurements using Cu Kα radiation (λ=1.5406 Å) on a Bruker D8 Advance diffractometer to determine their crystal structure. Morphological studies of the materials were conducted using field emission scanning electron microscopy (FESEM, JEOL, JSM-7600F), transmission electron microscopy (TEM, JEOL, JEM-2100F), and high-angle annular dark-field scanning transmission electron microscopy (HAADF-STEM, JEM-ARM300F) equipped with a probe corrector. Raman spectroscopy analysis was performed using a Renishaw Raman microscope with a laser wavelength of 532 nm. X-ray photoelectron spectroscopy (XPS) measurements were carried out on a Kratos Axis Supra electron spectrometer to analyze surface chemistry. To further investigate the internal structure of the materials, Brunauer-Emmett-Teller (BET) specific surface area and pore structure were evaluated at 77 K using a Micromeritics ASAP 2020 system. The local structure of the obtained catalysts was analyzed by measuring Ni/Fe K-edge X-ray absorption near-edge structure (XANES) and extended X-ray absorption fine structure (EXAFS) data at the 10 C beamline of the Pohang Accelerator Laboratory (PAL, Pohang, South Korea). The energy of the measured spectra was calibrated using the reference spectra of elemental Co and Fe foils, which were collected simultaneously.

**S1.2 Electrochemical measurements**

All electrochemical tests were conducted at room temperature using a standard three-electrode system on an electrochemical workstation (760E, CH Instruments). The half-cell comprised a platinum foil counter electrode, an Ag/AgCl (3 M KCl solution) reference electrode, and a working electrode prepared by drop-casting catalyst ink onto a glassy carbon (GC) substrate. To prepare the catalyst ink, 5 mg of each catalyst was dispersed in a mixed solution containing 1 mL of ethanol/water (1:1, v/v) and 25 μL of 5 wt% Nafion solution, followed by ultrasonication at low temperature for 30 minutes to form a homogeneous black suspension. The catalyst ink was then drop-cast onto freshly polished GC electrode surfaces and dried in ambient air.

The ORR performance of the catalysts was measured in O_2_-saturated 0.1 M KOH solution. Cyclic voltammetry (CV) curves were obtained at a scan rate of 50 mV s^-1^. Linear sweep voltammetry (LSV) was conducted using a rotating disk electrode (RDE) or a rotating ring-disk electrode (RRDE) at a scan rate of 5 mV s^-1^ with rotation speeds of 400, 625, 900, 1225, and 1600 rpm. The ORR stability of the electrocatalysts was also performed by chronoamperometry at a potential of 0.60 V (vs. RHE) in O_2_-staurated 0.1 M KOH. Simultaneously, the methanol tolerance test is conducted during the chronoamperometric measurements by injecting methanol into the electrolyte and observing the changes in current density. In addition, The OER performance of the catalysts was also measured in 0.1 M KOH solution and the LSV was performed at a scan rate of 5 mV s^-1^ with a rotation speed of 1,600 rpm. The OER-LSV polarization curves have been corrected by subtracting the background current in O_2_-saturated 0.1 M KOH solution. All potentials were calibrated to the standard reversible hydrogen electrode (RHE) using the Nernst equation, E_RHE_=E_Ag/AgCl_+0.21+0.0592×pH. The half-wave potential (E_1/2_) is defined as the electrode potential at which the current density equals half of the limiting current density. The overpotential (E_j=10_) is defined as the potential required to achieve a current density of 10 mA cm^-^². The number of transferred electrons was calculated using the Koutecky-Levich (K-L) equation:

$\frac{1}{J}=\frac{1}{J_{k}}+\frac{1}{J_{L}}=\frac{1}{J_{k}}+\frac{1}{B\omega^{1/2}}$ (S1)

$B=0.62nFC_{0}{D_{0}}^{2/3}\nu^{-1/6}$ (S2)

where 𝐽 is the measured current density, 𝐽_𝑘_ is the kinetic diffusion current density, 𝐽_𝐿_ is the kinetic limiting current density, 𝐵 is the reciprocal of the slope, ω is the angular velocity (ω=2πN, where N is the rotation speed), 𝑛 is the number of transferred electrons, 𝐹 is the Faraday constant (96485 C mol^-1^), 𝐶_0_ is the saturation concentration of O_2_ (1.2×10^-6^ mol cm^-3^), 𝐷_0_ is the diffusion coefficient of O_2_ (1.9×10^-5^ cm^2^ s^-1^), and ν is the dynamic viscosity (0.01 cm^-2^ s^-1^).

RRDE measurements were conducted to investigate the four-electron selectivity of the as-prepared samples. The hydrogen peroxide (H_2_O_2_) yield and electron transfer number (n) were calculated by the following equations:

$H_{2}O_{2}(\%)=200\times\frac{\frac{I_{r}}{N}}{I_{d}+\frac{I_{r}}{N}}$ (S3)

$n=4\times\frac{I_{d}}{I_{d}+\frac{I_{r}}{N}}$ (4)

where *I_d_* is the disk current, *I_r_* is the ring current, and *N* is the current collection efficiency of the platinum ring (N=0.37).

**S1.3 Computational methods**

All the spin-polarized first principle density functional theory (DFT) calculations were performed using Vienna ab initio simulation package (VASP) with the projector augmented wave (PAW) method. The generalized gradient approximation (GGA) with the Perdew-Burke-Ernzerh (PBE) of exchange-functional was utilized to model the electron-ion interaction. The energy cutoff for the plane wave basis expansion was set to 520 eV and the force on each atom less than 0.02 eV/Å was set for convergence criterion of geometry relaxation. A 15 Å vacuum was added along the z direction in order to avoid the interaction between periodic structures. The structure model was constructed by a 8×8×1 supercell of graphene. Brillouin-zone integration was sampled by single Γ point. The self-consistent calculations apply a convergence energy threshold of 10^-5^ eV. The DFT-D3 method was employed to consider the van der Waals interaction.

The free energy of each elementary step in the proton coupled electron transfer reactions was computed using the computational hydrogen electrode (CHE) model for oxygen reduction reaction (ORR). Considering the O_2_ molecular is not broken before reduction, the associative 4e- reduction pathway was evaluated to be most feasible for ORR in this work, as follows:

O_2_* + H_2_O + e^−^→ OOH* + OH^−^ (S5)

OOH* + e^−^→ O* + OH^−^  (S6)

O* + H_2_O + e^−^ → OH* + OH^−^ (S7)

OH* + e^−^ →OH^−^ + * (S8)

where * represents the active site on the corresponding surface.

For the OER in this study, the pathway is as follows:

OH^−^ + *→ OH* + e^−^ (S9)

OH* + OH^−^ → O* + H_2_O + e^−^ (S10)

O* + OH^−^ → OOH* + e^−^ (S11)

OOH* + OH^−^ → O_2_* + H_2_O + e^−^ (S12)

where * represents the active site on the corresponding surface.

The free energies of the OER/ORR steps were calculated using the equation:

ΔG = ΔE_DFT_ + ΔE*_ZPE_* − TΔS (S13)

where ΔE_DFT_ is the DFT energy difference, and the ΔE_ZPE_ and TΔS terms were obtained based on vibration analysis.

**S1.4 Rechargeable Zn-air battery assembly and measurements**

The liquid Zn-air batteries (ZABs) were assembled by using polished zinc foil as the anode, aqueous solution containing 6 M KOH + 0.2 M Zn(CH_3_COO)_2_ as the electrolyte, and FeN_4_-Ru_6_-NiN_4_@PCA or commercial Pt/C+RuO_2_ coated on carbon paper as the air cathode. The open circuit voltage, discharge polarization curves, charge polarization curves, and voltage curves at different current densities were measured using a CHI 760E electrochemical workstation. For cyclic stability testing, the cathodes were prepared from FeN_4_-Ru_6_-NiN_4_@PCA. For comparison, Pt/C+RuO_2_ (mass ratio of 1/1) was also used to prepare the cathodes of ZABs. The constant current charge-discharge cyclic stability test of ZABs was conducted at room temperature using a Neware battery testing station system (CT-4008Tn), with a current density of 2 mA cm^-2^ (discharged for 10 minutes per cycle, followed by charging for 10 minutes).

The preparation method of the flexible self-supported air cathode is as follows: coat the uniform FeN_4_-Ru_6_-NiN_4_@PCA catalyst ink onto the flexible carbon paper membrane. Solid-state flexible rechargeable zinc-air batteries (ZABs) are assembled using polished zinc foil as the anode and PAW ternary hydrogel as the solid-state electrolyte. The galvanostatic charge-discharge cycle test of the flexible ZABs is conducted at a current density of 1 mA cm⁻² (10 minutes for discharge and 10 minutes for charge). The preparation steps of PAW are as follows: 4g of acrylamide, 4 mg of N,N'-methylenebisacrylamide, and 10 mg of potassium persulfate were dissolved in 7 mL of Milli-Q water and 3 mL of DMSO under magnetic stirring for 2 h, obtaining a homogenous solution. The result ant solution was poured into a transparent box. Then the box was sealed with tape and heated at 60 ℃ to trigger the free-radical polymerization for 12 h. Soak the film in an alkaline solution containing 6 M potassium hydroxide (KOH) and 0.2 M zinc acetate (Zn(CH₃COO)₂) for 24 hours to obtain the PAW ternary hydrogel.

**S2 Supplementary Figures and Tables**


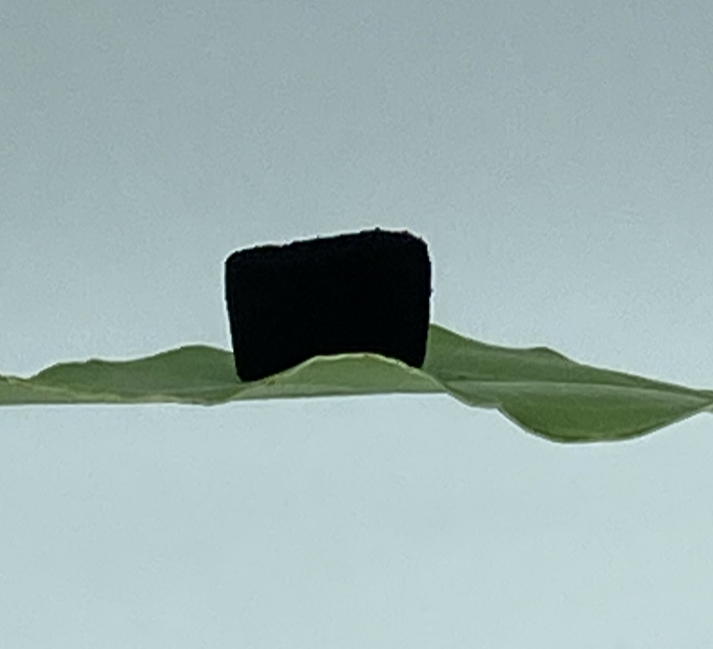


**Fig. S1** Morphologies of aerogels after carbonization of FeN_4_-Ru_6_-NiN_4_@PCA


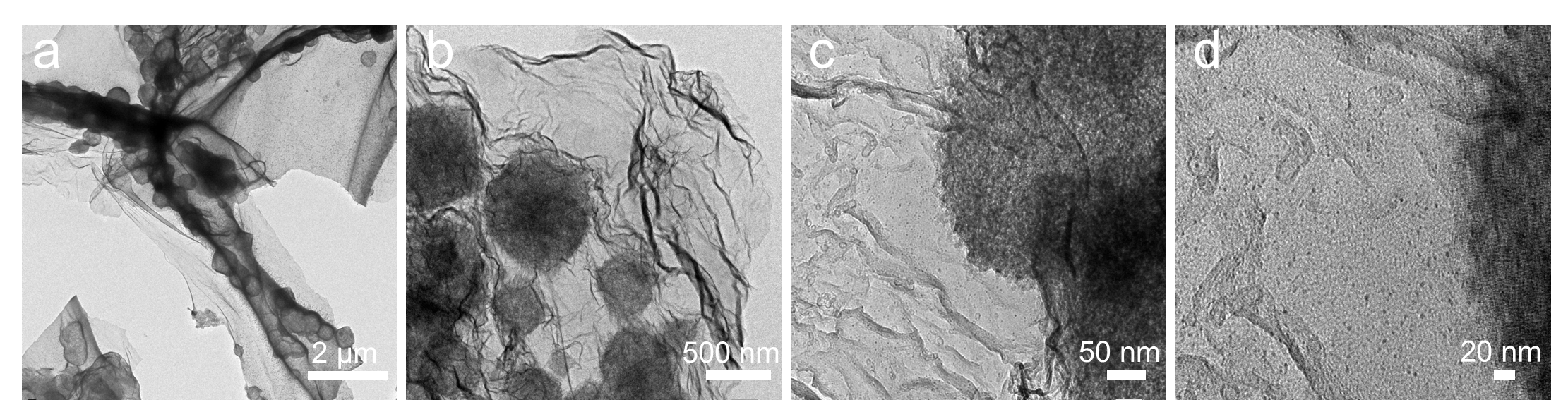


**Fig. S2** (a,b,c,d) TEM of FeN_4_-Ru_6_-NiN_4_@PCA


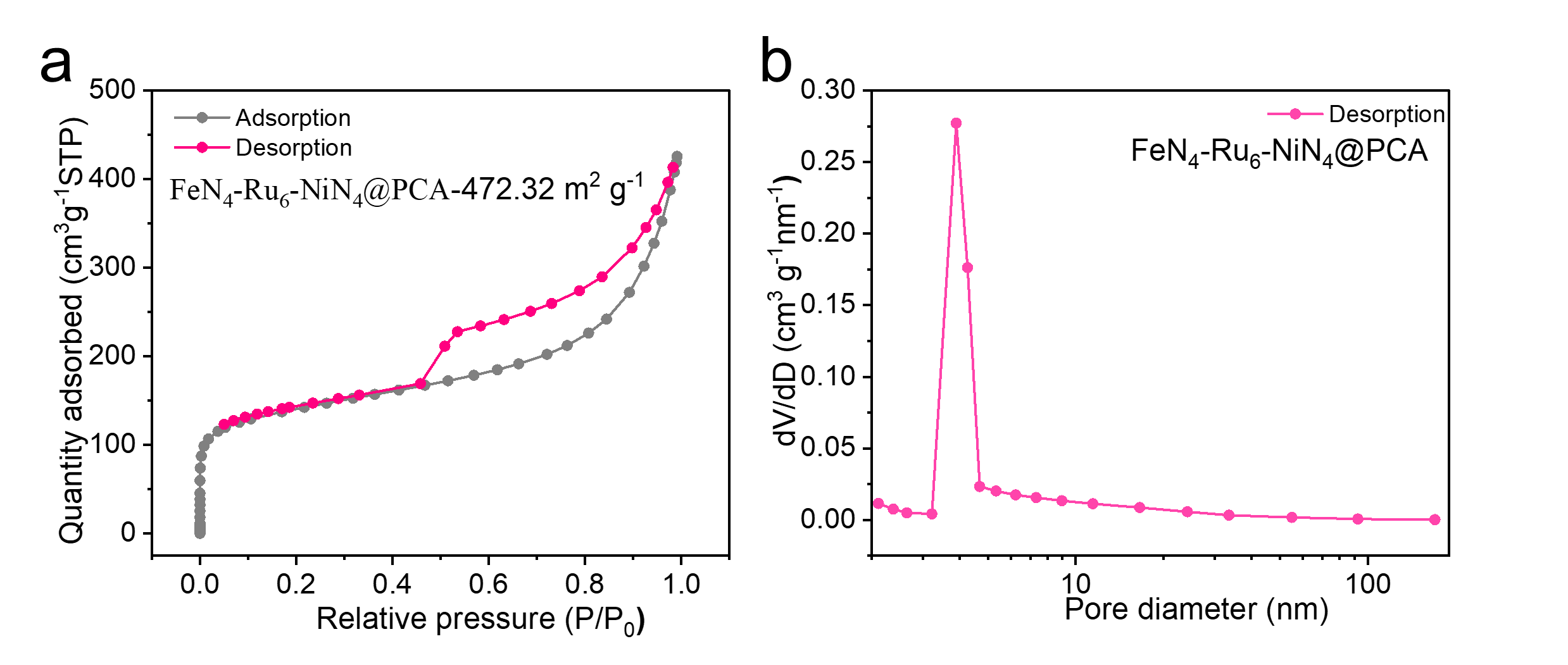


**Fig. S3** (a) N_2_ adsorption-desorption isotherms of FeN_4_-Ru_6_-NiN_4_@PCA, (b) the corresponding pore size distribution


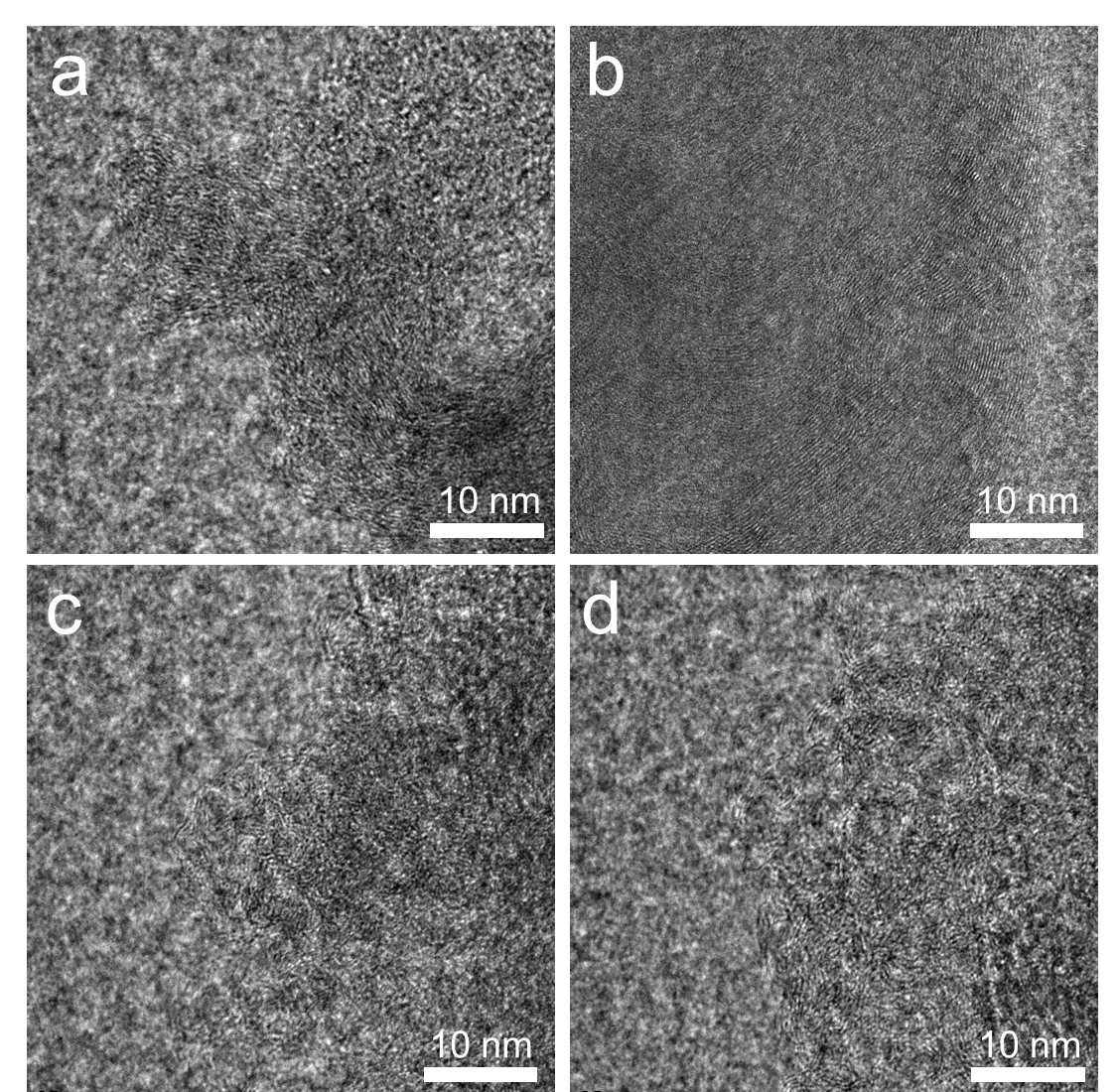


**Fig. S4** (a,b,c,d) HR-TEM data of FeN_4_-Ru_6_-NiN_4_@PCA


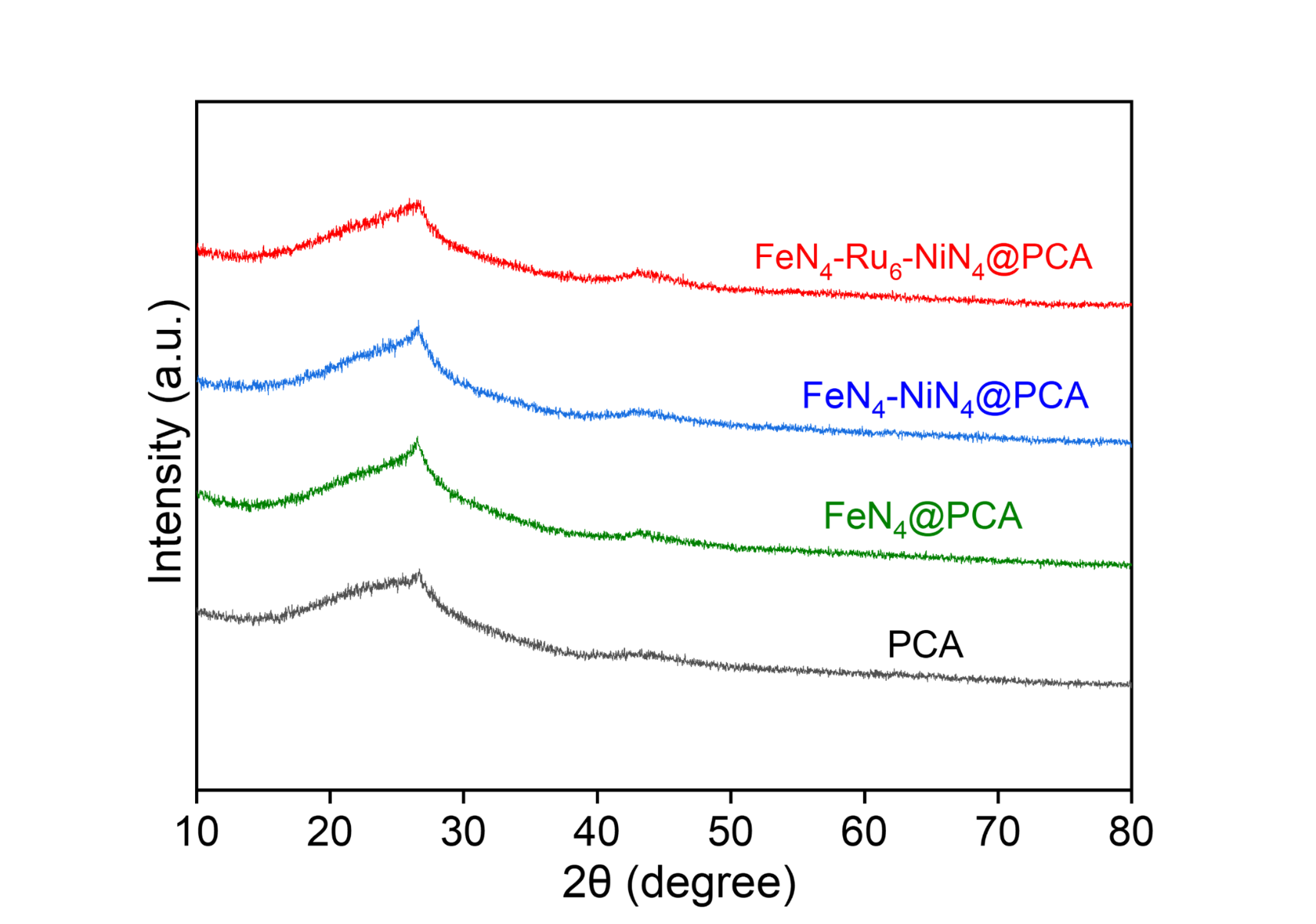


**Fig. S5** (a) XRD patterns of PCA, FeN_4_@PCA, FeN_4_-NiN_4_@PCA, and FeN_4_-Ru_6_-NiN_4_@PCA

**
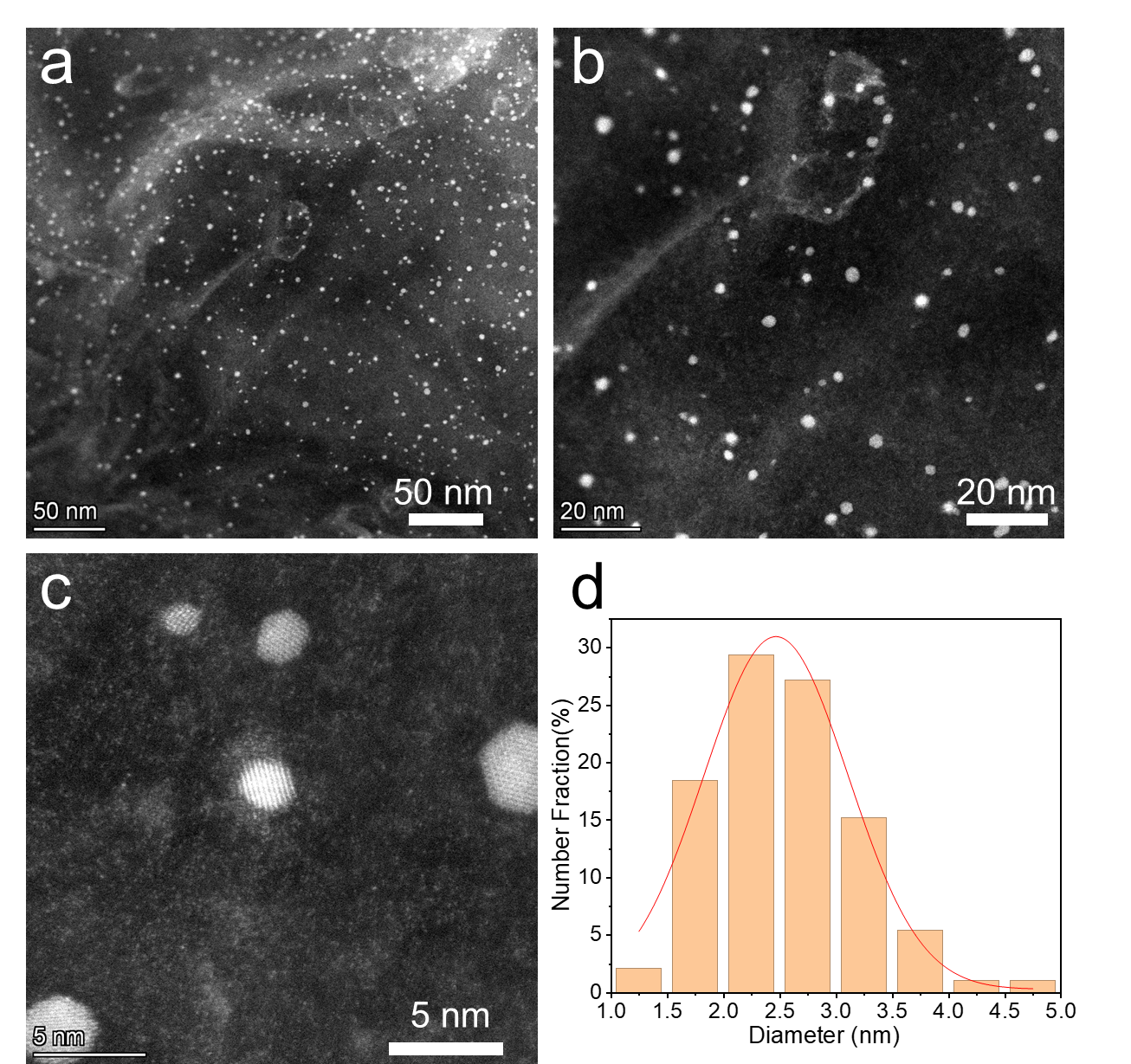
**

**Fig. S6.** (a,b,c) AC-STEM images of FeN_4_-Ru_6_-NiN_4_@PCA and (d) Particle size distribution diagram of Ru nanoclusters

**
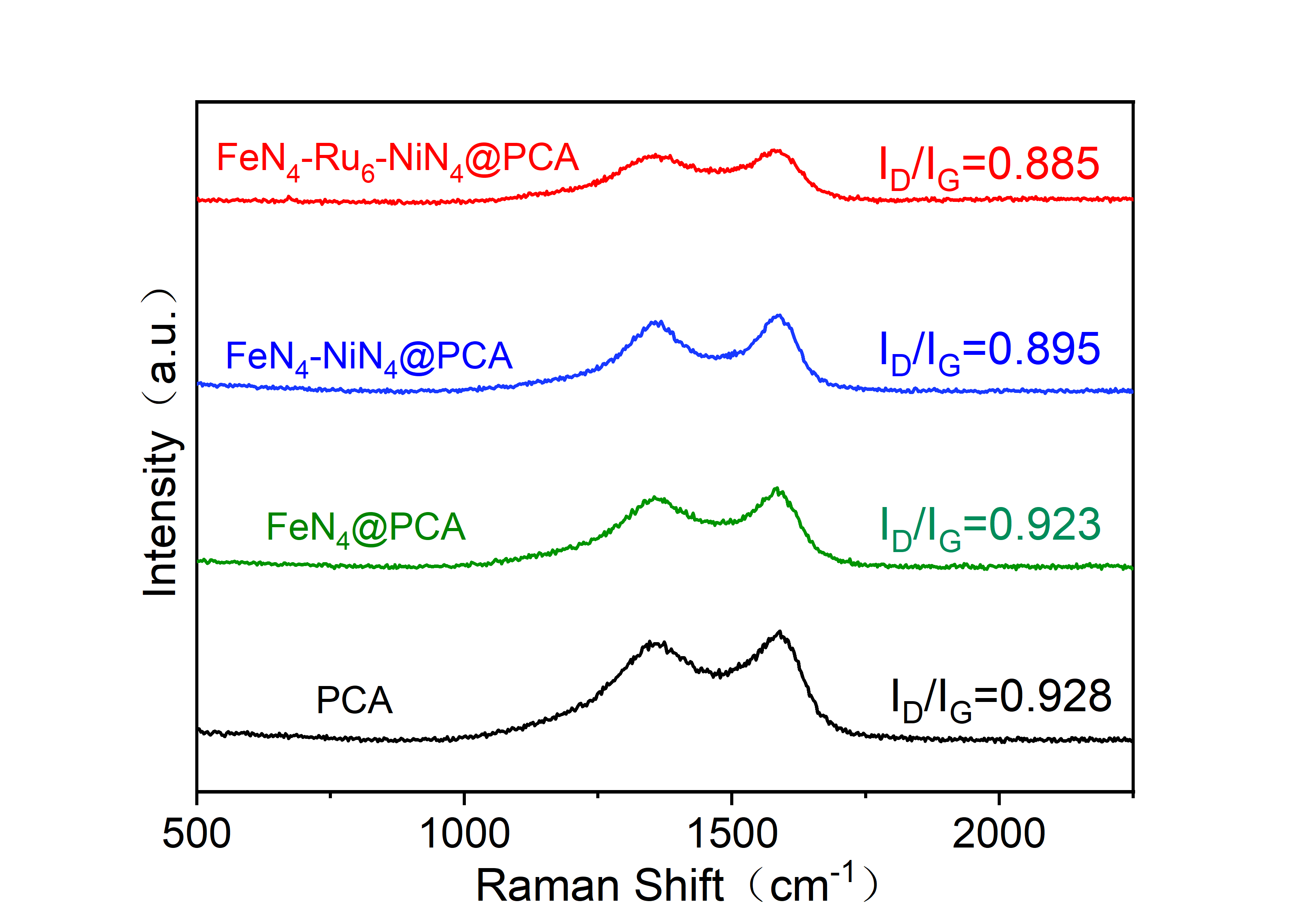
**

**Fig. S7** Raman spectra of PCA, FeN_4_@PCA, FeN_4_-NiN_4_@PCA, and FeN_4_-Ru_6_-NiN_4_@PCA


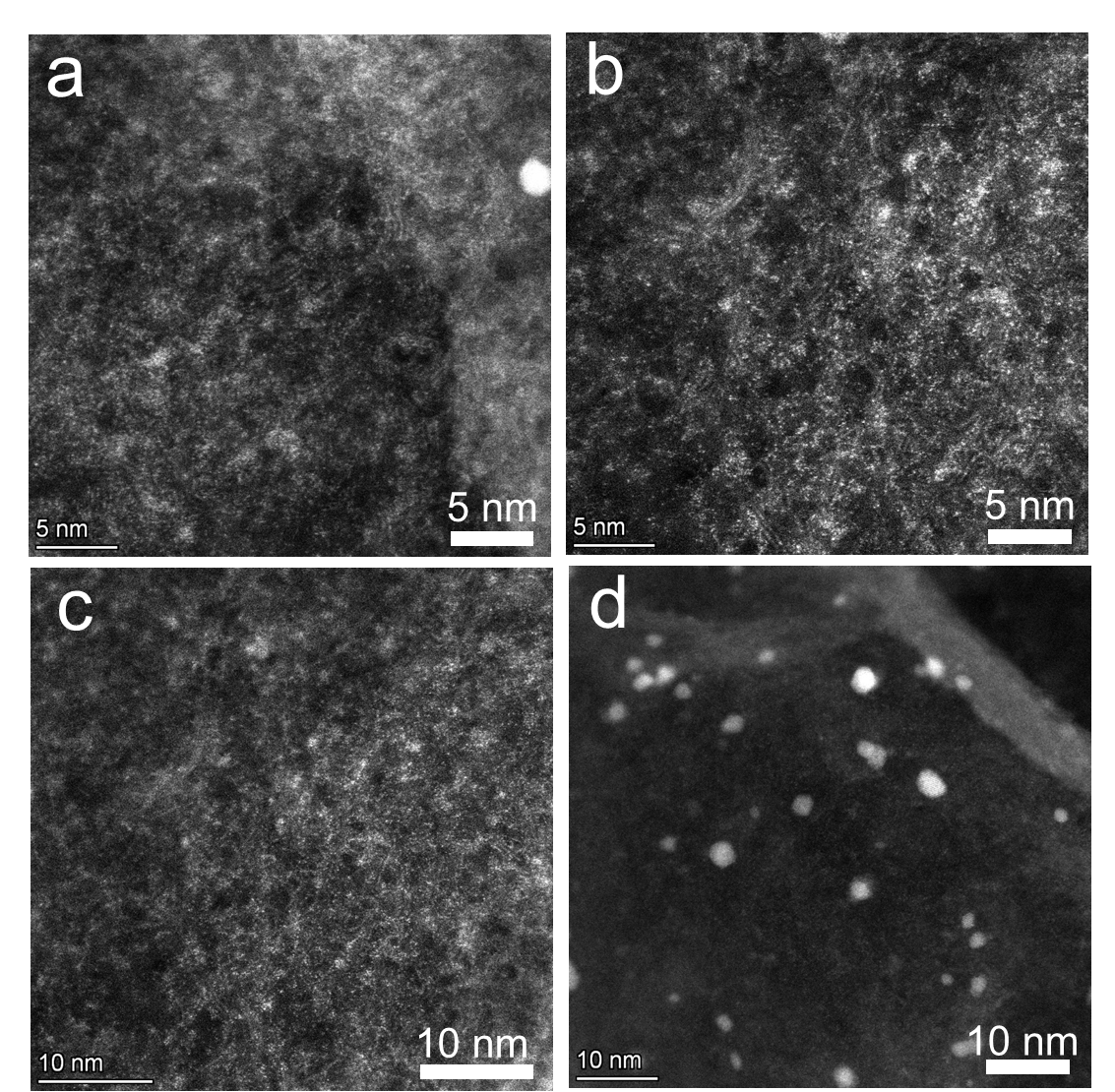


**Fig. S8** (a,b,c,d) AC-STEM images of FeN_4_-Ru_6_-NiN_4_@PCA


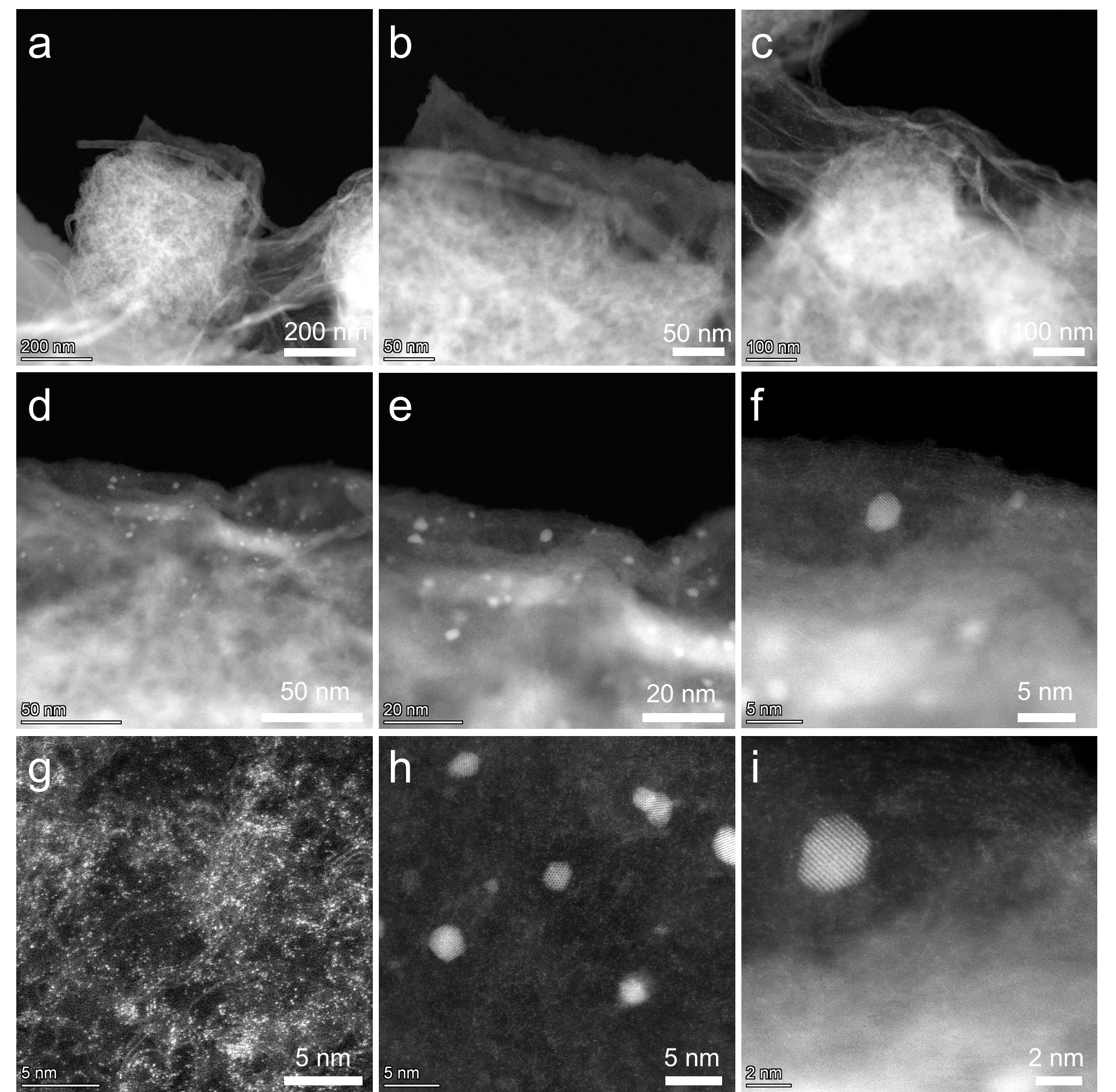


**Fig. S9** (a,b,c,d,e,f,g,h,i) AC-STEM images of FeN_4_-Ru_6_-NiN_4_@PCA


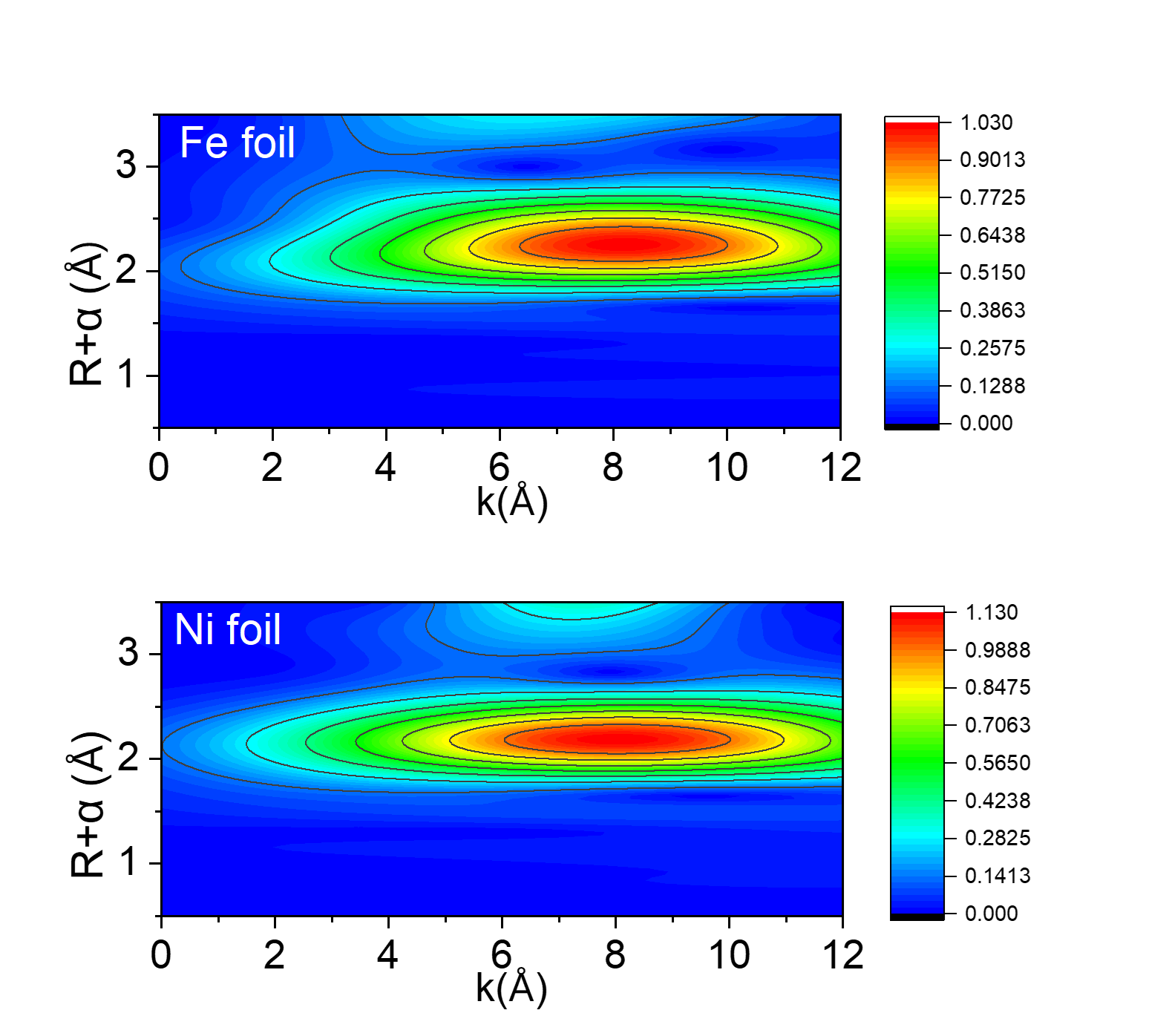


**Fig. S10** WT data of the *k*^3^-weighted EXAFS spectra of Fe foil


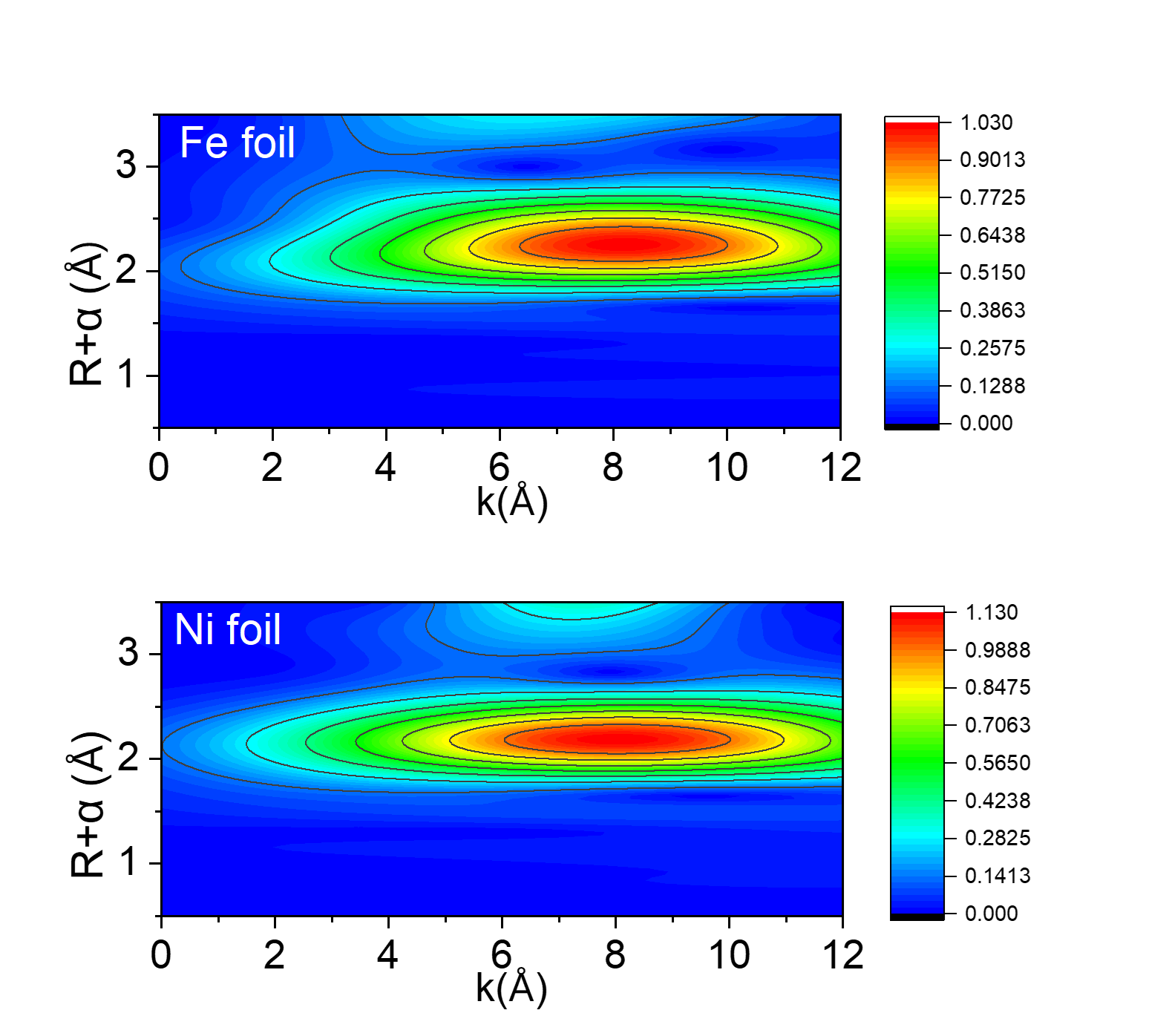


**Fig. S11** WT data of the *k*^3^-weighted EXAFS spectra of Ni foil


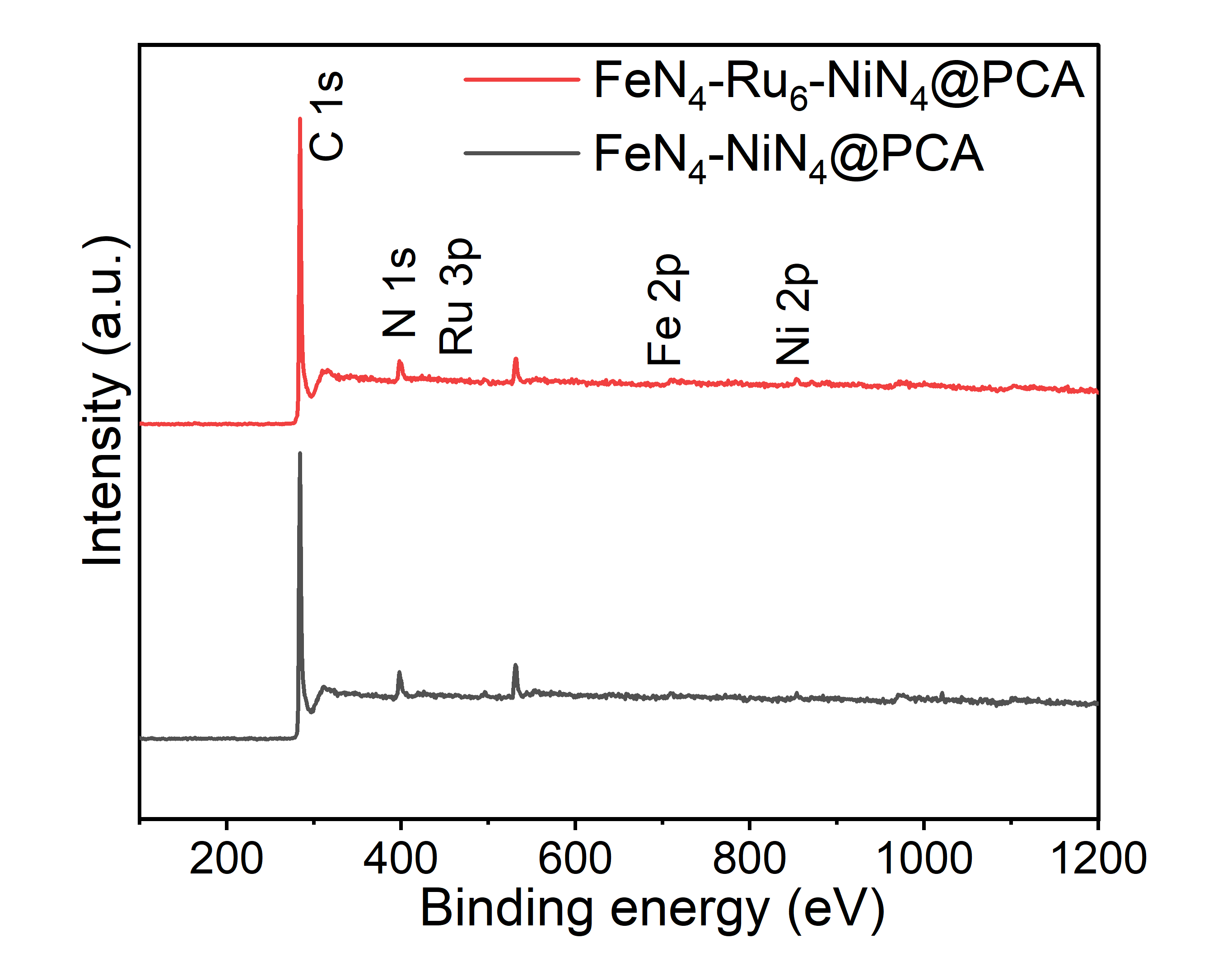


**Fig. S12.** The full XPS survey spectra of FeN_4_-Ru_6_-NiN_4_@PCA and FeN_4_-NiN_4_@PCA


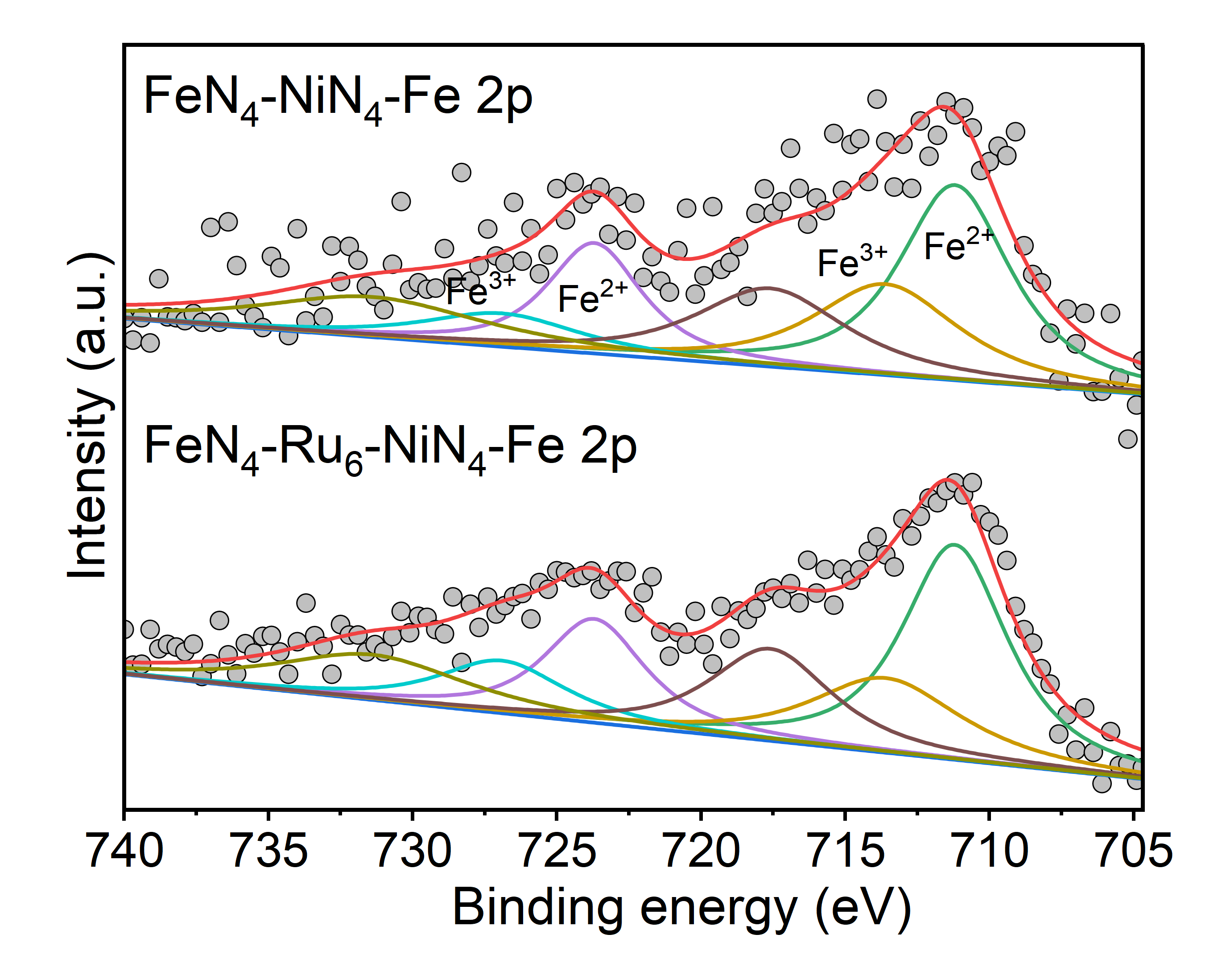


**Fig. S13** The high-resolution XPS Fe 2p spectra of FeN_4_-Ru_6_-NiN_4_@PCA and FeN_4_-NiN_4_@PCA


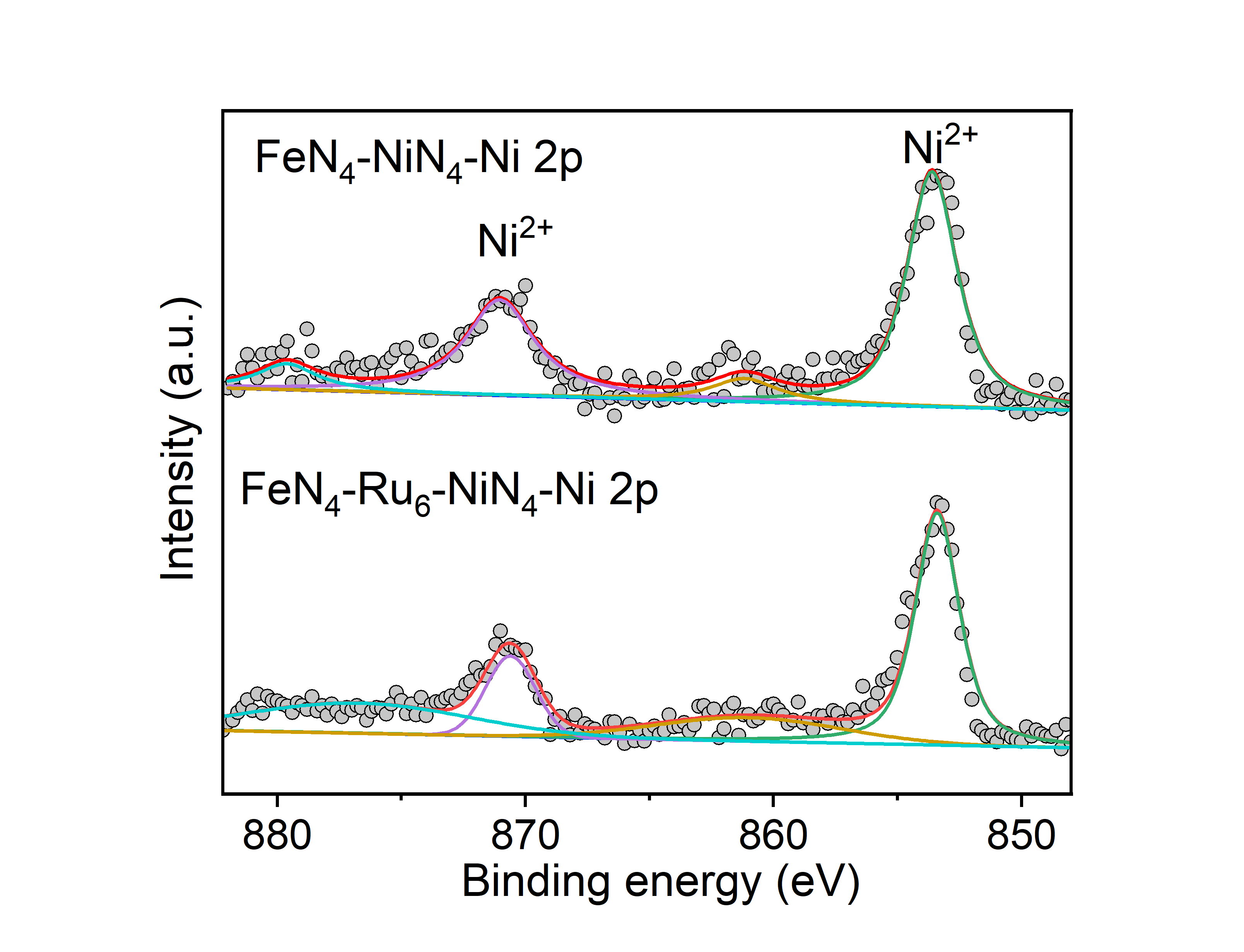


**Fig. S14** The high-resolution XPS Ni 2p spectra of FeN_4_-Ru_6_-NiN_4_@PCA and FeN_4_-NiN_4_@PCA

**
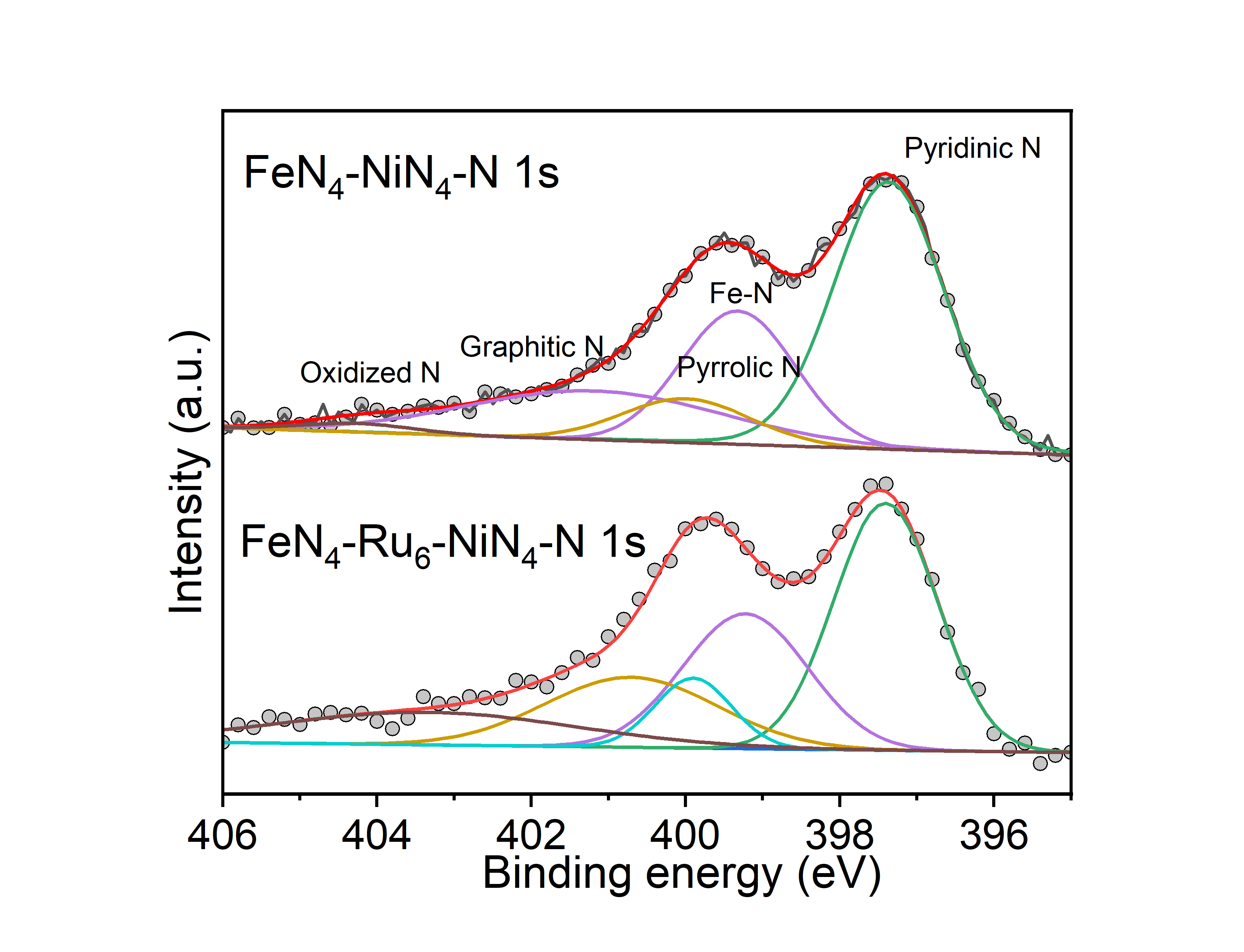
**

**Fig. S15** High-resolution XPS N 1s spectra of FeN_4_-Ru_6_-NiN_4_@PCA and FeN_4_-NiN_4_@PCA


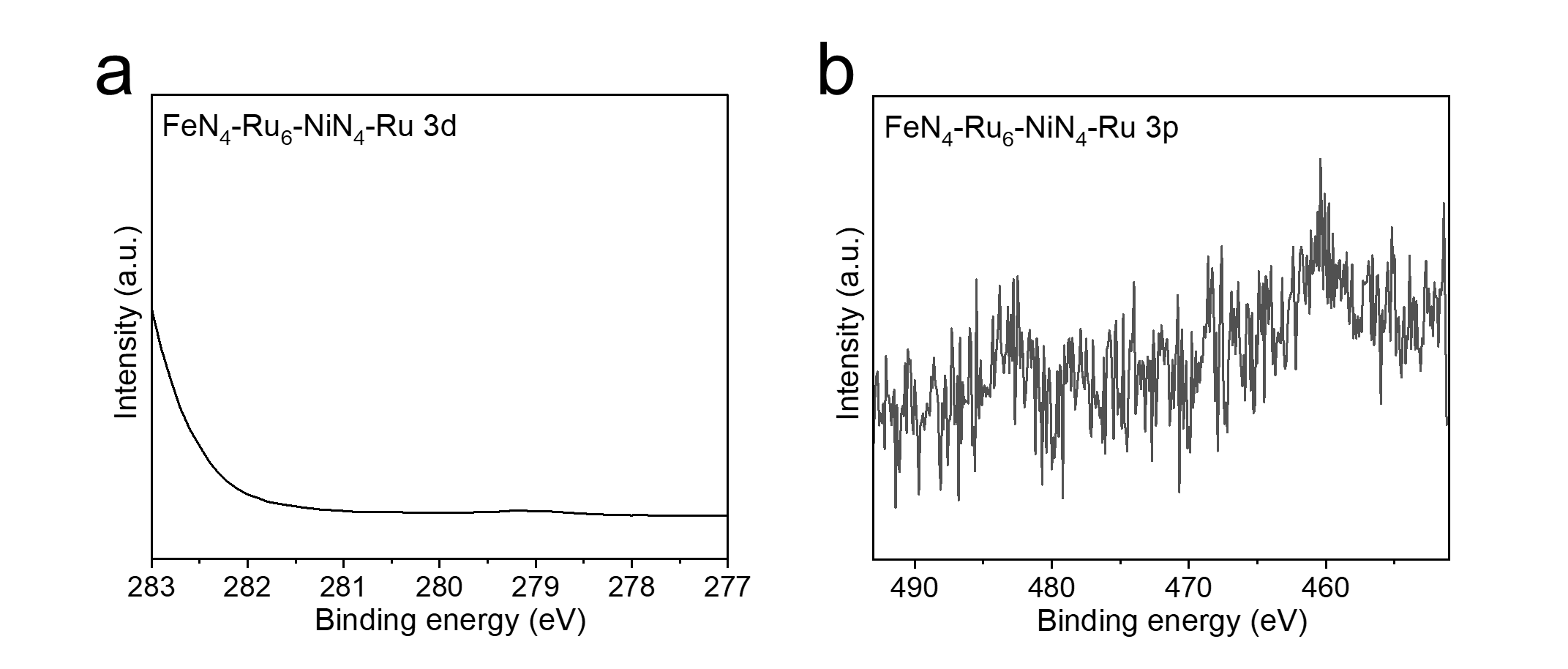


**Fig. S16** High-resolution XPS Ru 3d and 3p spectra of FeN_4_-Ru_6_-NiN_4_@PCA

**
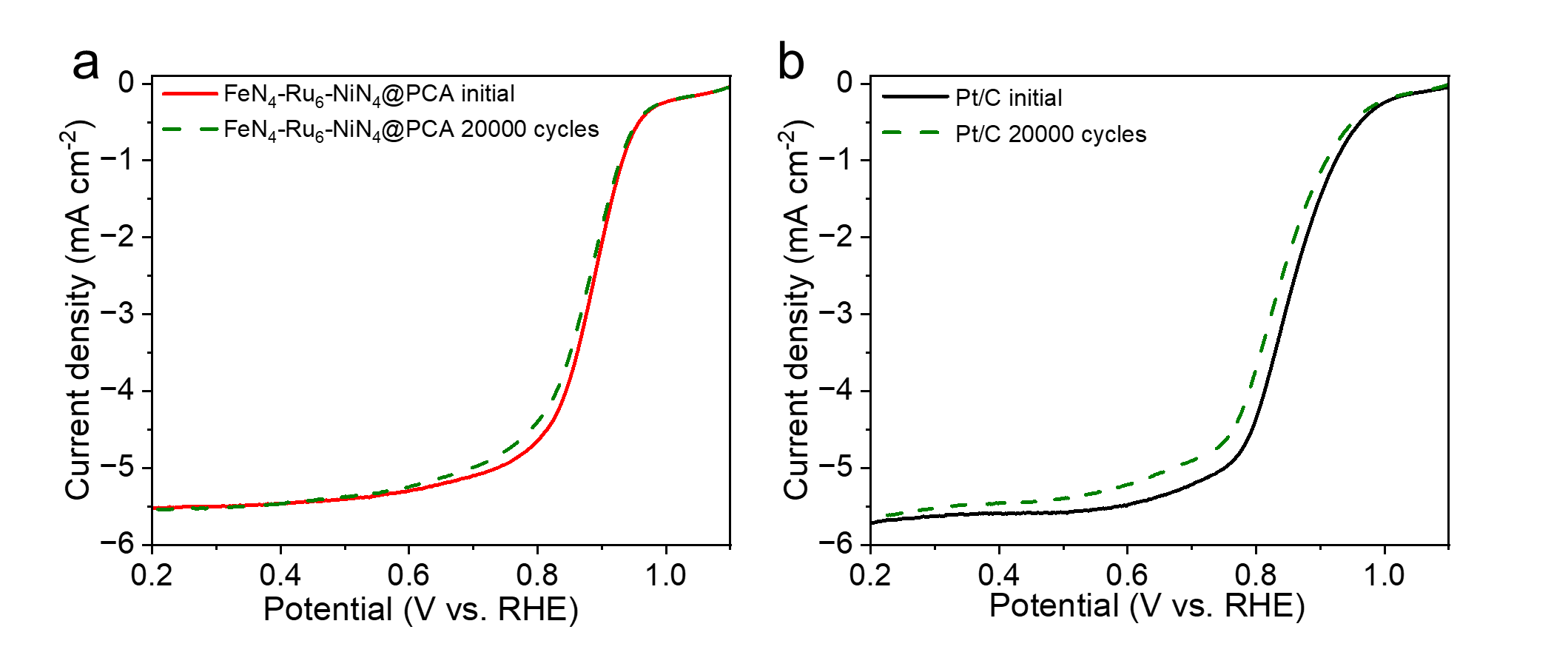
**

**Fig. S17** ORR LSV curves of FeN_4_-Ru_6_-NiN_4_@PCA and Pt/C after 20000 potential cycles


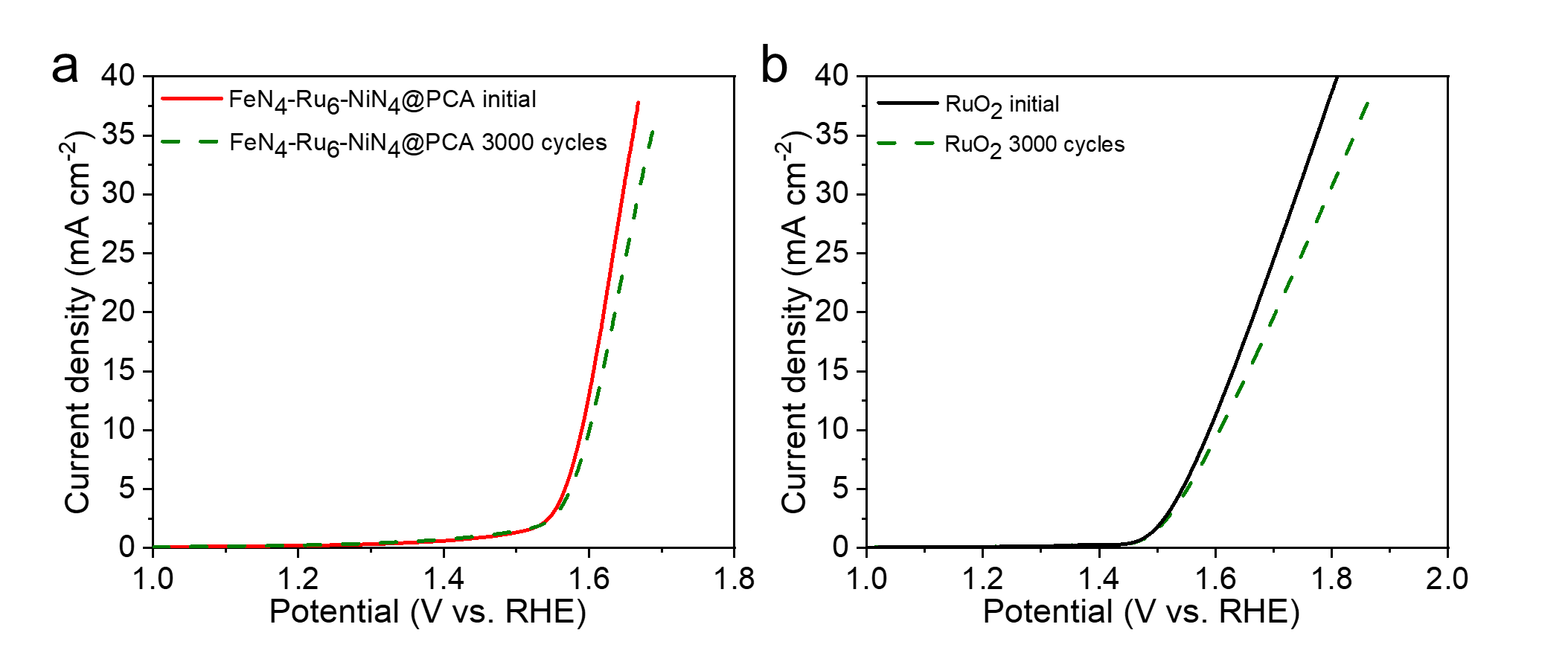


**Fig. S18** OER LSV curves of FeN_4_-Ru_6_-NiN_4_@PCA and RuO_2_ after 3000 potential cycles


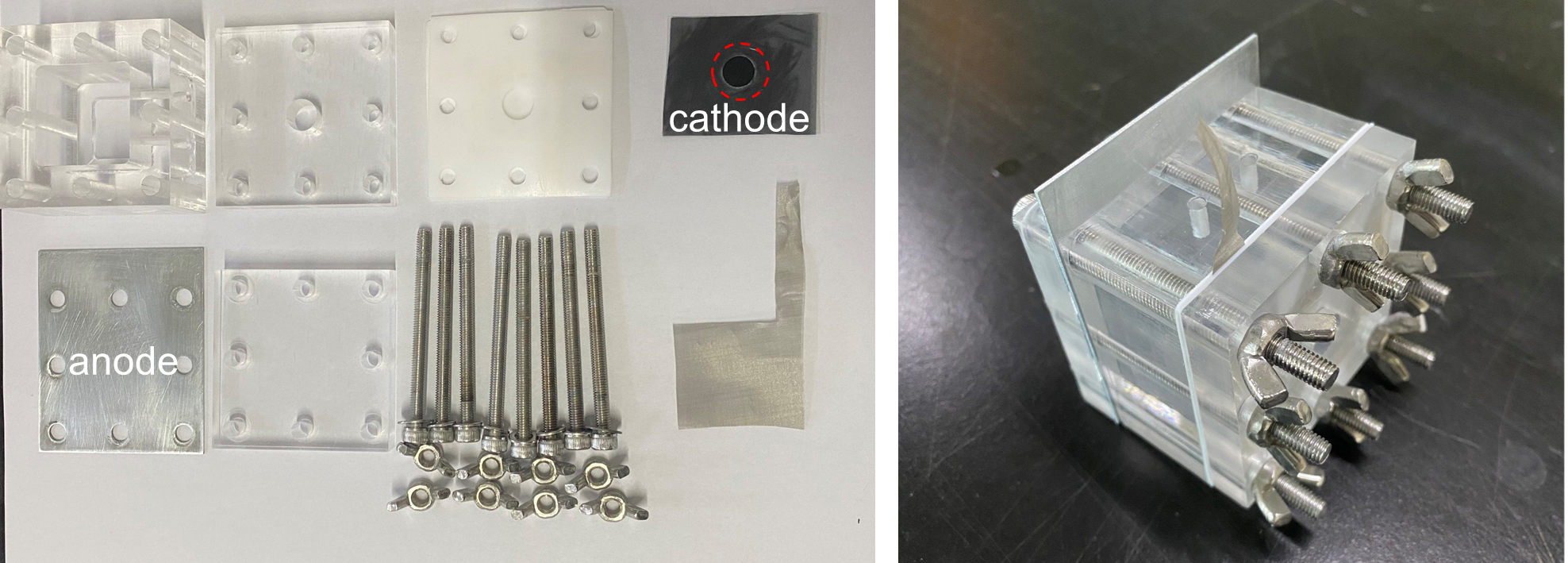


**Fig. S19** Digital photo of the assembled rechargeable liquid Zn-air battery


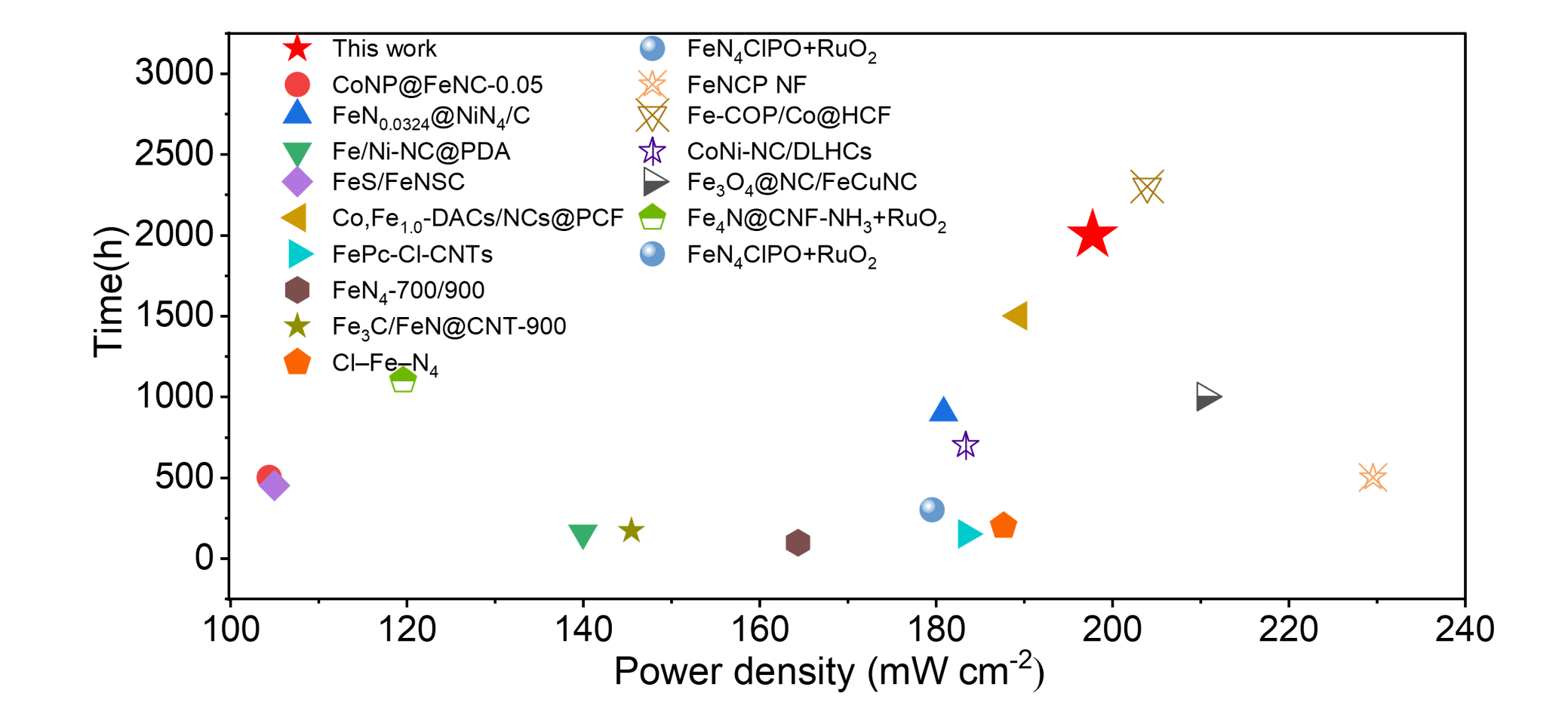


**Fig. S20** Comparison of the performance of rechargeable liquid Zn-air batteries reported in recent literatures


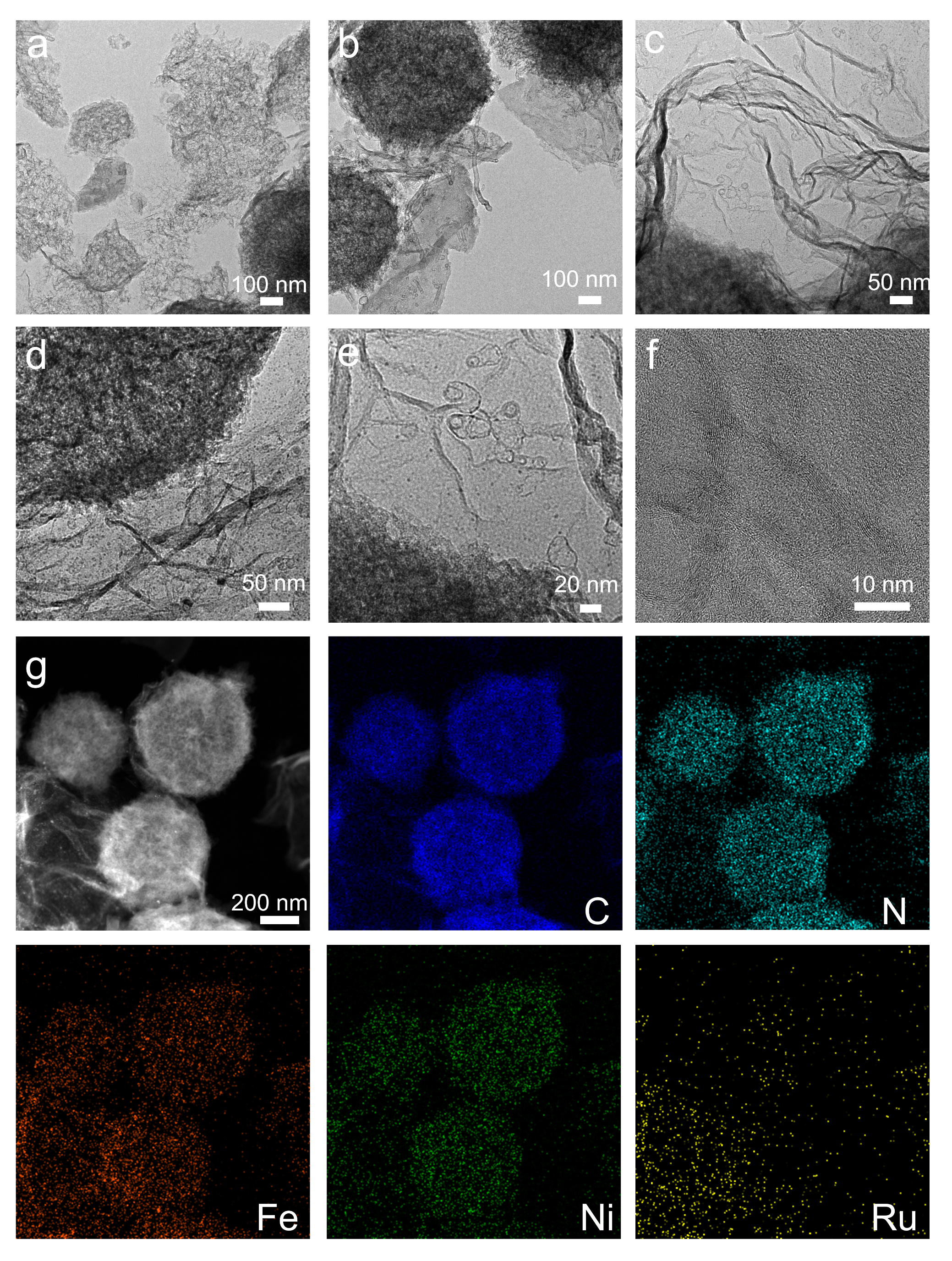


**Fig. S21** (a,b,c,d,e,f) TEM and EDS elemental mapping of FeN_4_-Ru_6_-NiN_4_@PCA after cycling stability test


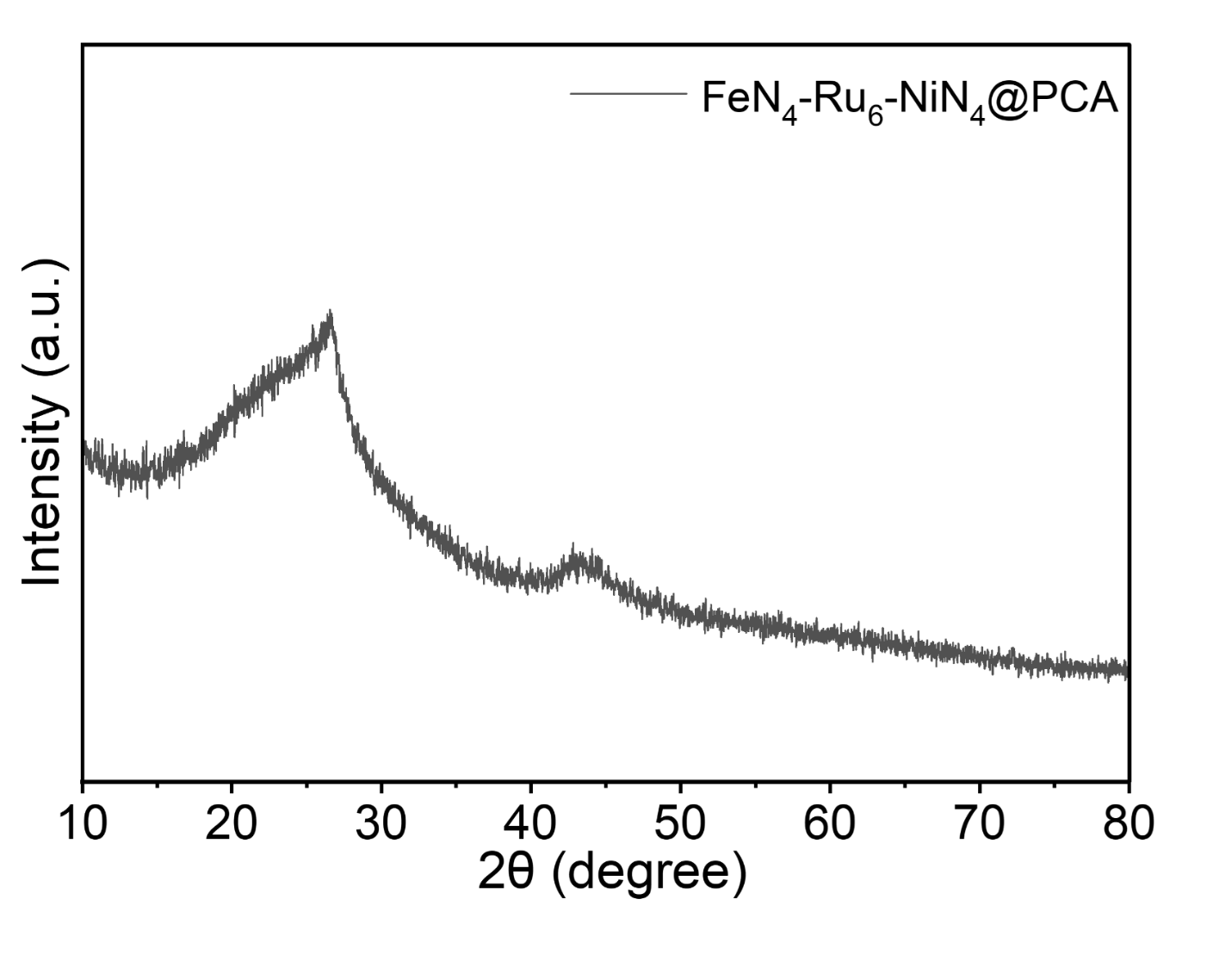


**Fig. S22** XRD pattern of FeN_4_-Ru_6_-NiN_4_@PCA after cycling stability test

**
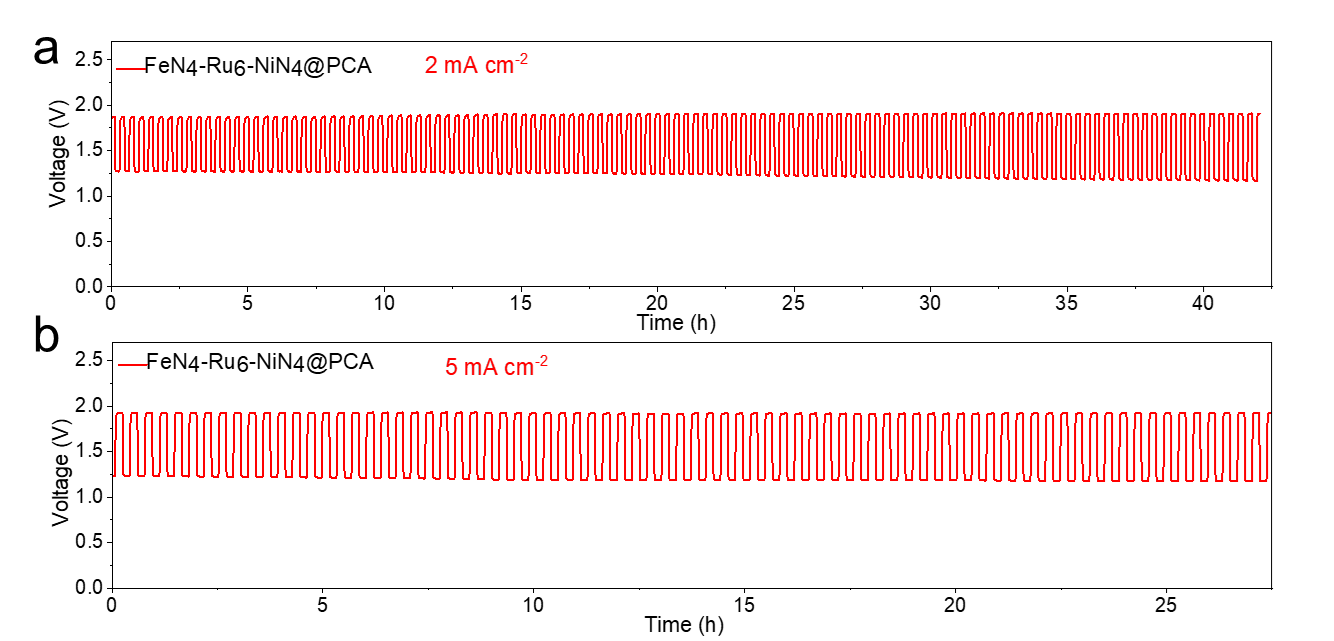
**

**Fig. S23** Constant current charge–discharge cycle performance of the flexible solid-state ZABs at 2 mA cm^-2^ and 5 mA cm^-2^


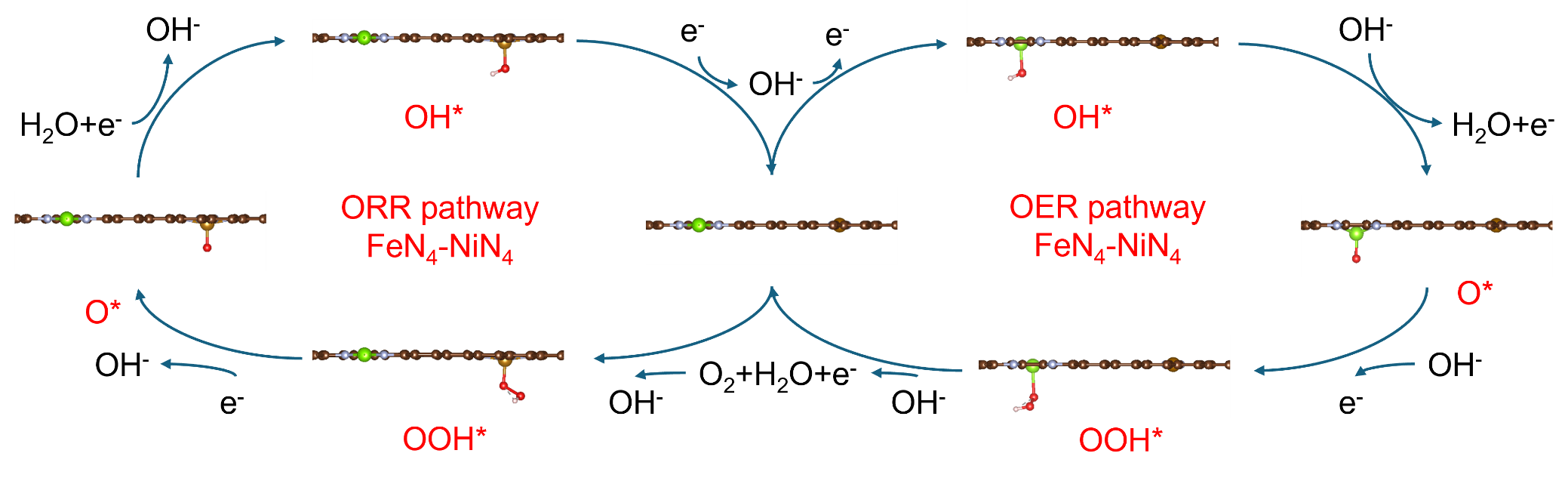


**Fig. S24** Adsorption configurations at each key step on the FeN_4_ sites in the FeN_4_-NiN_4_ model

**Table S1** The Ni K-edge,Fe K-edge and Ru K-edge EXAFS curves fitting parameters in FeN_4_-Ru_6_-NiN_4_@PCA

Note: R is the interatomic distance (the bond length between central atoms and surrounding coordination atoms), σ^2^ is Debye-Waller factor (a measure of thermal and static disorder in absorber-scatter distances).

**Table S2** ICP-OMS of FeN_4_-Ru_6_-NiN_4_@PCA

| **Sample** | **ICP-OMS (wt. %)** | | |
| --- | --- | --- | --- |
|  | **Fe** | **Ni** | **Ru** |
| **FeN_4_-Ru_6_-NiN_4_@PCA** | **0.82** | **0.74** | **0.39** |

**Table S3** Comparison of bifunctioal ORR and OER catalytic properties of FeN_4_-Ru_6_-NiN_4_@PCA and control samples

| Catalyst materials | ORR | OER | ΔE |
| --- | --- | --- | --- |
| **FeN_4_-Ru_6_-NiN_4_@PCA** | **0.874** | **1.58** | **0.706** |
| PCA | 0.75 | 1.72 | 0.970 |
| FeN_4_@PCA | 0.864 | 1.72 | 0.856 |
| FeN_4_-NiN_4_@PCA | 0.860 | 1.63 | 0.77 |
| Pt/C | 0.851 | - | - |
| RuO_2_ | - | 1.59 | - |
